# Deep Learning‐Assisted Rapid Bacterial Classification Based on Raman Spectroscopy of Bacteria Lysed by Acoustically Driven Fiber‐Tip Vibration

**DOI:** 10.1002/advs.202507724

**Published:** 2025-07-08

**Authors:** Yukai Liu, Miaomiao Ji, Xiao Ren, Zhenyong Dong, Tian Wen, Qingyue Dong, Ho‐pui Ho, Lunbiao Cui, Yanqing Lu, Guanghui Wang

**Affiliations:** ^1^ Key Laboratory of Intelligent Optical Sensing and Integration of the Ministry of Education College of Engineering and Applied Sciences Nanjing University Nanjing Jiangsu 210023 P. R. China; ^2^ NHC Key laboratory of Enteric Pathogenic Microbiology Jiangsu Provincial Medical Key Laboratory of Pathogenic Microbiology in Emerging Major Infectious Diseases Jiangsu Provincial Center for Disease Control and Prevention Nanjing Jiangsu 210009 P. R. China; ^3^ Department of Biomedical Engineering The Chinese University of Hong Kong Shatin Hong Kong SAR 999077 P. R. China

**Keywords:** acoustofluidic lysis, bacterial classification, deep learning, raman spectroscopy, vibrating fiber‐tip

## Abstract

Rapid and accurate identification of bacterial pathogens is critical for effective clinical decision‐making and combating antibiotic resistance. Surface‐enhanced Raman spectroscopy (SERS) combined with machine learning (ML) offers a powerful method for rapid, label‐free bacterial identification. Conventional methods rely on surface molecular structures for identification, yet the richer and unique spectral information from intracellular biomolecules is often masked by the bacterial envelope, limiting classification accuracy. Here, a novel bacterial classification method is demonstrated by introducing acoustofluidic lysis based on the vibrating fiber‐tip, combined with Raman spectroscopy and deep learning. The fiber‐tip oscillates in a torsional mode, generating a controlled single‐vortex within a capillary to concentrate bacteria in high‐shear regions, enhancing lysis efficiency. This process effectively exposes intracellular components such as nucleic acids, proteins, and lipids, significantly enhancing the expression of features in bacterial Raman spectra, improving both spectral resolution and information richness. A residual neural network (ResNet) model is further employed for automated classification, achieving 98.9% accuracy across seven bacterial samples, surpassing traditional classifiers like random forests. The clinical validation experiments highlight the method's potential for real‐world applications, enabling direct, on‐site detection of clinical samples and facilitating rapid diagnostics, thus offering a promising advancement in pathogen identification.

## Graphical abstract

A novel acoustofluidic lysis technique based on a vibrating fiber‐tip is integrated with Raman spectroscopy and deep learning for rapid, label‐free bacterial classification. By exposing intracellular biomolecules, this method significantly enhances spectral resolution, enabling high‐accuracy classification with a 1D‐ResNet model. The approach offers a promising tool for clinical diagnostics and real‐time pathogen identification.

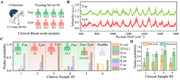

## 1 Introduction

Each year, an estimated 7.7 million deaths worldwide are attributed to bacterial infections, emphasizing the growing severity of this global health crisis.^[^ ^1^ ^]^ Rapid and accurate identification of pathogens is urgently needed to facilitate timely clinical decision‐making and targeted treatments in the early stages of infection, helping to curb the spread of infections and improve patient outcomes.^[^ ^2^ ^]^ However, traditional bacterial detection methods such as mass spectrometry ^[^ ^3^ ^]^, culture‐based techniques ^[^ ^4^, ^5^ ^]^, and polymerase chain reaction (PCR)^[^ ^6^ ^]^ are limited by high costs, complex pre‐treatment, and time‐consuming procedures, making it difficult to satisfy the demand for rapid diagnostics in modern healthcare settings.^[^ ^7^ ^]^

Surface‐enhanced Raman spectroscopy (SERS)^[^ ^8^ ^]^ offers a powerful analytical tool due to its rapid, sensitive, and label‐free capabilities.^[^ ^9^, ^10^ ^]^ SERS can provide detailed structural information at the molecular level, making it effective for online qualitative analysis of complex samples, such as bacterial detection.^[^ ^11^ ^]^ The unique molecular compositions of each bacterial strain can be characterized by a distinct fingerprint in spectra, which can be used for precise identification.^[^ ^12^ ^]^ However, traditional assessment methods rely on artificial experience to analyze and identify spectral features, which is highly subjective and poorly certain, limiting clinical applications.^[^ ^13^, ^14^ ^]^ Fortunately, the integration of machine learning (ML), particularly deep learning (DL) techniques, provides a solution to these challenges.^[^ ^15^ ^]^ By automating data processing and leveraging advanced pattern recognition, DL extracts complex features from extensive spectra with minimal human intervention, enhancing both efficiency and precision.^[^ ^16^ ^]^ This combination significantly expands the practical applications of Raman spectroscopy by improving its analytical capabilities and delivering faster, more reliable results.^[^ ^17^ ^]^

Nonetheless, the performance of ML‐assisted spectral classification remains highly sensitive to noise and background interference, making it heavily reliant on the quality and diversity of bacterial Raman spectra.^[^ ^18^ ^]^ Currently, acquiring high‐quality spectra is challenging due to the limitations imposed by the bacterial envelope.^[^ ^19^ ^]^ First, the bacterial envelope's strong scattering limits light penetration, resulting in spectra dominated by surface components and masking internal molecular signals.^[^ ^20^ ^]^ Second, the bacterial envelope prevents metal nanoparticles needed for SERS enhancement from reaching the interior, limiting signal amplification.^[^ ^21^ ^]^ Third, the high similarity in bacterial envelopes often leads to overlapping spectral features, compromising spectral quality and classification specificity.^[^ ^22^ ^]^ In conclusion, the presence of a bacterial envelope fundamentally limits the acquisition of high‐quality full‐fingerprint spectra, thereby impacting classification performance. Therefore, using lysis to disrupt the bacterial envelope and expose intracellular components is essential for enhancing spectral differentiation and classification specificity.^[^ ^23^ ^]^ Traditional lysis techniques, such as mechanical pulverization^[^ ^24^ ^]^, are equipment‐dependent and cumbersome, while chemical^[^ ^25^, ^26^ ^]^ and enzymatic^[^ ^27^, ^28^ ^]^ lysis are time‐consuming and lack versatility. In addition, these methods lack the ability to consistently focus or enrich bacteria at the microscale, leading to incomplete or uneven lysis.^[^ ^29^ ^]^ In contrast, the acoustofluidic methods offer a promising alternative to concentrate and lyse bacteria in a rapid, efficient, and mild manner.^[^ ^30^, ^31^ ^]^

In this work, we introduce a novel acoustofluidic lysis technique to enhance bacterial classification by integrating Raman spectroscopy with deep learning. The acoustofluidic component employs a pulled quartz fiber actuated by acoustic waves. Due to its low mass and stiffness, the sharp fiber resonates readily under low‐frequency excitation and oscillates periodically in controlled modes, with each mode generating a distinct streaming pattern. Within a confined capillary, we demonstrated the enrichment of particles from micrometer to nanometer scale, using a tornado‐like, 3D single‐vortex induced by a torsional vibration. This vortex continually draws the bacteria into regions of peak vibration and flow velocity, where they are exposed to intense, multiple stresses (especially strong shear), causing them to rupture. This rupture enables intracellular biomolecules to interact directly with silver nanoparticles, enhancing their expression in Raman spectra and improving both spectral richness and resolution. After applying simple acoustofluidic processing across seven biological samples (six bacterial species and one mammalian cell line), we collected the spectral datasets with different levels of lysis. We then use a random forest classifier for rapid classification from these enhanced spectra. To further improve classification performance, we introduced a residual neural network (ResNet) mode, which demonstrated superior accuracy and robustness compared to traditional classifiers. In the future, we aim to rapidly predict not only species but also potential subspecies by combining our acoustofluidic technology with the well‐trained 1D‐ResNet model.

## 2 Results and Discussion

### 2.1 Conception of Rapid Acoustofluidic Lysis to Enhance Classification of Raman Spectra Assisted by Deep Learning

**Figure 1:**
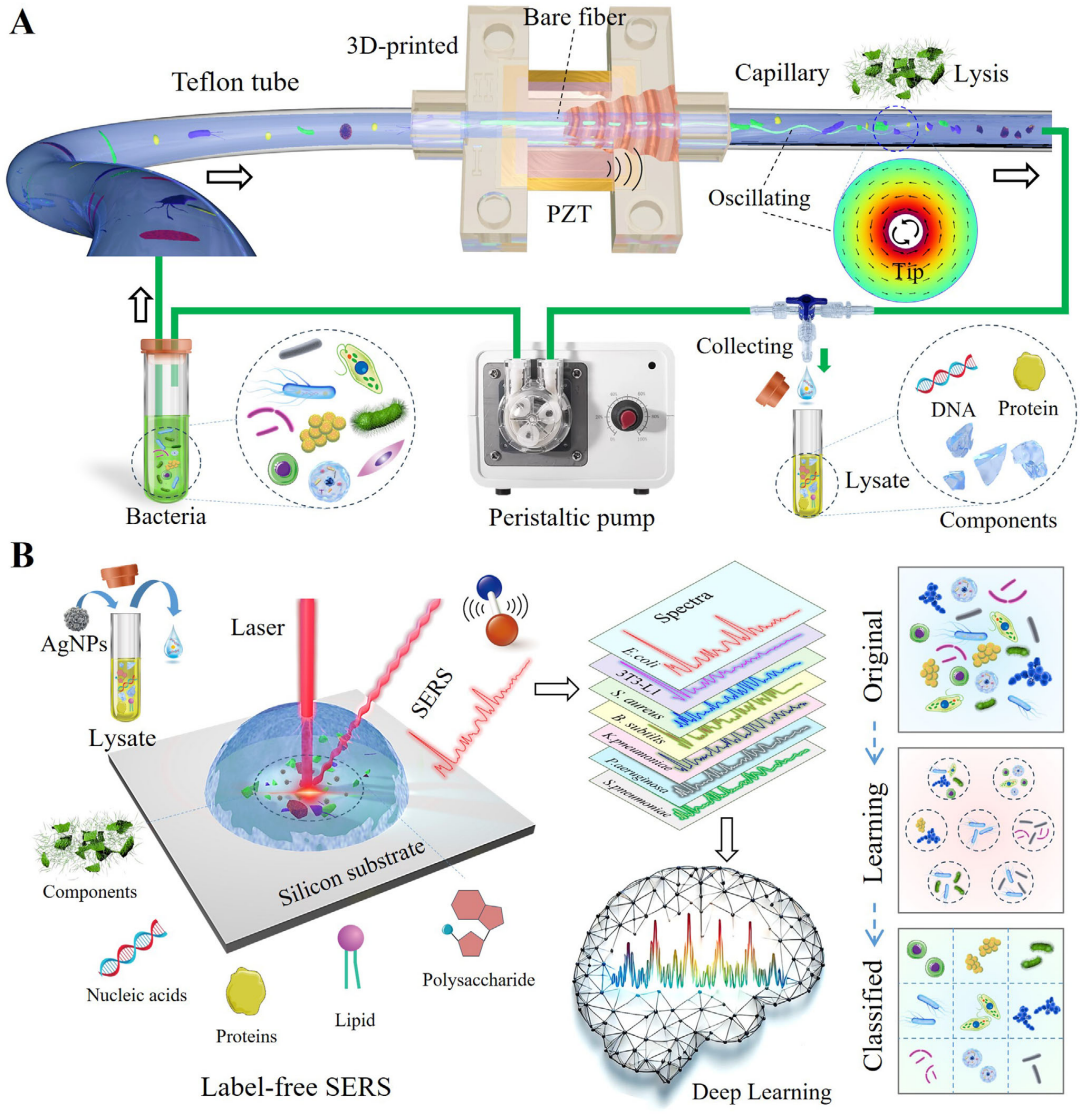
Schematic of acoustofluidic lysis of bacteria through a vibrating fiber‐tip, followed by label‐free rapid classification via Raman spectroscopy assisted by Deep learning. A) The acoustofluidic system is designed for rapid lysis of bacteria/cells. Samples are pumped into the capillary channel, where mechanical waves induce the fiber tip to oscillate in a specific mode. This oscillatory mechanism generates high‐intensity vortices within the confined capillary, leading to the rupture of bacteria/cells and the exposure of intracellular components. B) Deep learning facilitates rapid and automated classification of bacteria using SERS spectra. The exposure of intracellular macromolecules enhances the detection of the bacterial “fingerprint,” significantly increasing the intensity of SERS signals and the richness of spectral information. This change can significantly increase the diversity of datasets, improving training effectiveness and predictive accuracy.

The schematic of acoustofluidic lysis of bacteria through vibrating fiber‐tip, followed by label‐free rapid classification of Raman spectra assisted by ML, is illustrated in **Figure** **1**. The system consists of simple, low‐cost components (Figure 1A), including a highly‐efficient acoustofluidic device and a microfluidic circulation module driven by a peristaltic pump. At the heart of the acoustofluidic device is a pulled sharp fiber, which transmits vibrations generated by a piezoelectric transducer encapsulated in a 3D‐printed housing. Fluid circulation is maintained by a peristaltic pump, with a manual three‐way valve for controlling sample circulation and collection. The fiber‐tip resonates with acoustic waves of a specific frequency and oscillates in a controlled mode. A torsional vibration mode is actuated to create a controlled single‐vortex within a capillary, enabling bacteria to concentrate in high shear regions, thereby enhancing lysis efficiency. As shown in the conceptual diagram of Figure 1B, key intracellular components such as nucleic acids, proteins, lipids, and polysaccharides are effectively released after acoustofluidic lysis treatment. This release process directly enhances the feature differences and information richness in Raman spectra compared to direct measurements, which is extremely favorable to ML. Accordingly, a novel method for bacterial classification and identification is proposed in this study by combining acoustofluidic lysis technology, SERS spectra, and a cutting‐edge deep learning (DL) model. The DL model automatically recognizes and reinforces the feature differences in the diversity spectra after lysis, which dramatically enhances the reliability and accuracy of the classification and identification.

### 2.2 Physical Mechanism of the Vibrating Fiber‐Tip Device

In the groundbreaking 2022 study by Daniel Ahmed and his team, they designed and implemented the first robotic arm effector assisted by the vibrating fiber‐tip, effectively showcasing the potential of this technology in diverse applications such as suction, fusion, particle capture, and mixing of viscous fluids.^[^ ^32^ ^]^ In subsequent research conducted in 2024, they achieved precise rotational control of zebrafish larvae, validating the stability and reproducibility of fiber‐tip vibration technology for fine manipulations.^[^ ^33^ ^]^ Building on these pioneering efforts, we have systematically expanded and deepened the research. We developed comprehensive numerical models to simulate vibrational modes and the resulting flow field distributions, and successfully demonstrated 3D manipulation of particles within capillaries. These advancements provide substantial support for further research and applications.

#### 2.2.1 Vibration and Acoustic Streaming at Fiber Tip

##### Vibration at Fiber Tip

**Figure 2:**
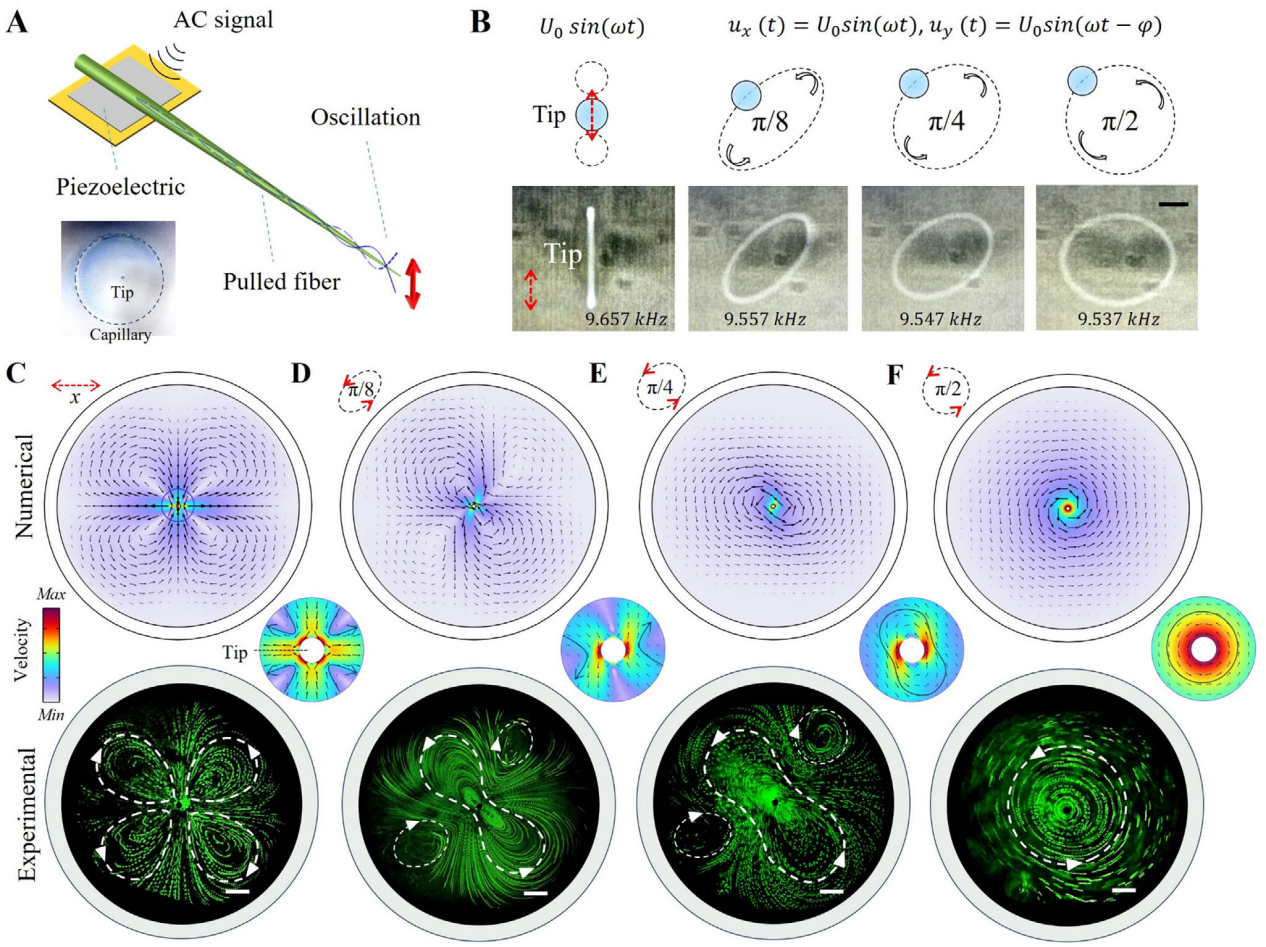
Working modes of the vibrating fiber‐tip and acoustic streaming induced at the tip. A) Acoustofluidic component employing a pulled fiber actuated by a piezoelectric transducer. The tapered structure efficiently transmits mechanical waves along the pulled fiber, amplifying vibrations toward the tip. B) Various vibration modes at the tip of the fiber. The vibration trajectory can be decomposed into *x*‐ and *y*‐direction displacements. A unidirectional vibration results in a linear trajectory, while counterclockwise elliptical trajectories are formed when the vibration in the *y*‐direction lags 𝜑 = π/8, π/4, and π/2 with respect to the vibration in the *x*‐direction. Scale bar: 100 µm. Numerical and experimental results of acoustic streaming patterns generated by the vibrating tip: C) Unidirectional *x*‐axis vibration; D) *y*‐axis vibration lagging the *x*‐axis by a phase of π/8; E) phase lag of π/4; F) phase lag of π/2. Scale bar: 100 µm.

The vibration of the transducer induces mechanical waves in the fiber, which propagate along the axis and are continuously amplified as the fiber tip tapers from thick to thin (**Figure** **2**A). The underlying mechanism of tip amplitude amplification can be explained by two key factors: energy focusing and the resonance effect. First, the fiber's gradual transition from a thicker to thinner structure functions as an energy guide during wave propagation, causing the mechanical wave energy to progressively converge toward the tip. This results in a sharp increase in energy density at the tip, significantly amplifying the vibration amplitude. Second, due to the tip's small mass and lower relative stiffness, its natural vibrational frequency is inherently low, making it easily resonant with external excitations. Thus, when the vibration frequency generated by the transducer approaches the tip's intrinsic frequency, a strong resonance occurs. This resonance not only facilitates the localized accumulation of vibrational energy but also markedly amplifies the tip's vibration amplitude, further enhancing the overall effect.

As shown in Figure 2B, an optical observation method was employed to capture the dynamic vibration behavior of the fiber tip under excitation. Specifically, a light source is incident at the distal end of the fiber, causing the tip to form a distinguishable bright spot under camera capture, which greatly improves the visualization of its vibration state. In the experiments, a lower excitation frequency (∼9.5 kHz) and high voltage amplitude (5 VPP) were applied, with the fiber positioned in a low‐damping air environment to maximize the vibration amplitude and improve visualization. The observations revealed two primary vibration modes at the fiber tip: linear and elliptical polarization vibration (named after the shape of the vibration trajectory). Under unidirectional excitation (e.g., along the *y*‐axis), the vibration followed a straight‐line trajectory in the direction of the applied force. However, when vibration occurs in two orthogonal directions (e.g., *x*‐ and *y*‐axes) with a phase difference (e.g., the *y*‐axis vibration lags the *x*‐axis by π/8, π/4, π/2, as demonstrated here), the vibration trajectory is transformed into closed elliptical paths. This transformation underscores the complex and polarized characteristics of the vibration. Additionally, both vibration modes exhibit frequency dependence. Video S1 (Supporting Information) provides visual evidence of the dynamic evolution of fiber‐tip vibration mode as the frequency varied from 9.45 to 9.8 kHz, captured from both front and top views.

##### 2.2.1.1 Acoustic Streaming at Fiber‐Tip

The acoustic streaming (AS) is primarily attributable to the action of the viscous boundary layer (Stokes layer) ^[^ ^34^, ^35^ ^],^ which is induced by the viscous shear force near the vibrating surface. The AS exhibits complex vortex patterns in regions outside the boundary layer, which are subject to regulation by various factors, including fluid boundary conditions, background flow field, vibration frequency, and amplitude. With vibration amplitudes at the fiber tip comparable to its own size, significant nonlinear effects arise, limiting the effectiveness of traditional perturbation methods. Accordingly, we employ finite element simulation (FEM) to examine the far‐field acoustic streaming (AS) generated by a tip oscillator within circular and rectangular capillaries (Figure S1, Supporting Information), with a particular focus on the circular capillary due to its symmetrical boundaries. To visualize the influence of tip vibration on AS within confined spaces, we placed the tip inside a 1 mm diameter glass capillary and employed digital particle image velocimetry (PIV) to track polystyrene microbeads, thereby visualizing the streaming. Figure 2C–F illustrate the intricate AS patterns simulated and experimented in diverse oscillation modes, and the insets zoom in on the AS details near the vibrating surface. The comparison results demonstrate that the FEM simulations align closely with the PIV experimental outcomes (see Video S2, Supporting Information for more details). The analysis indicates that variations in AS vortex patterns result from distinct vibration modes at the fiber tip, offering insights into understanding complex acoustofluidic behavior. By designing specific vibration modes, stable and controllable streaming patterns can be created, supporting further optimization of the acoustofluidic operation system.

#### 2.2.2 3D Particle Manipulation Along the Fiber‐Tip

**Figure 3:**
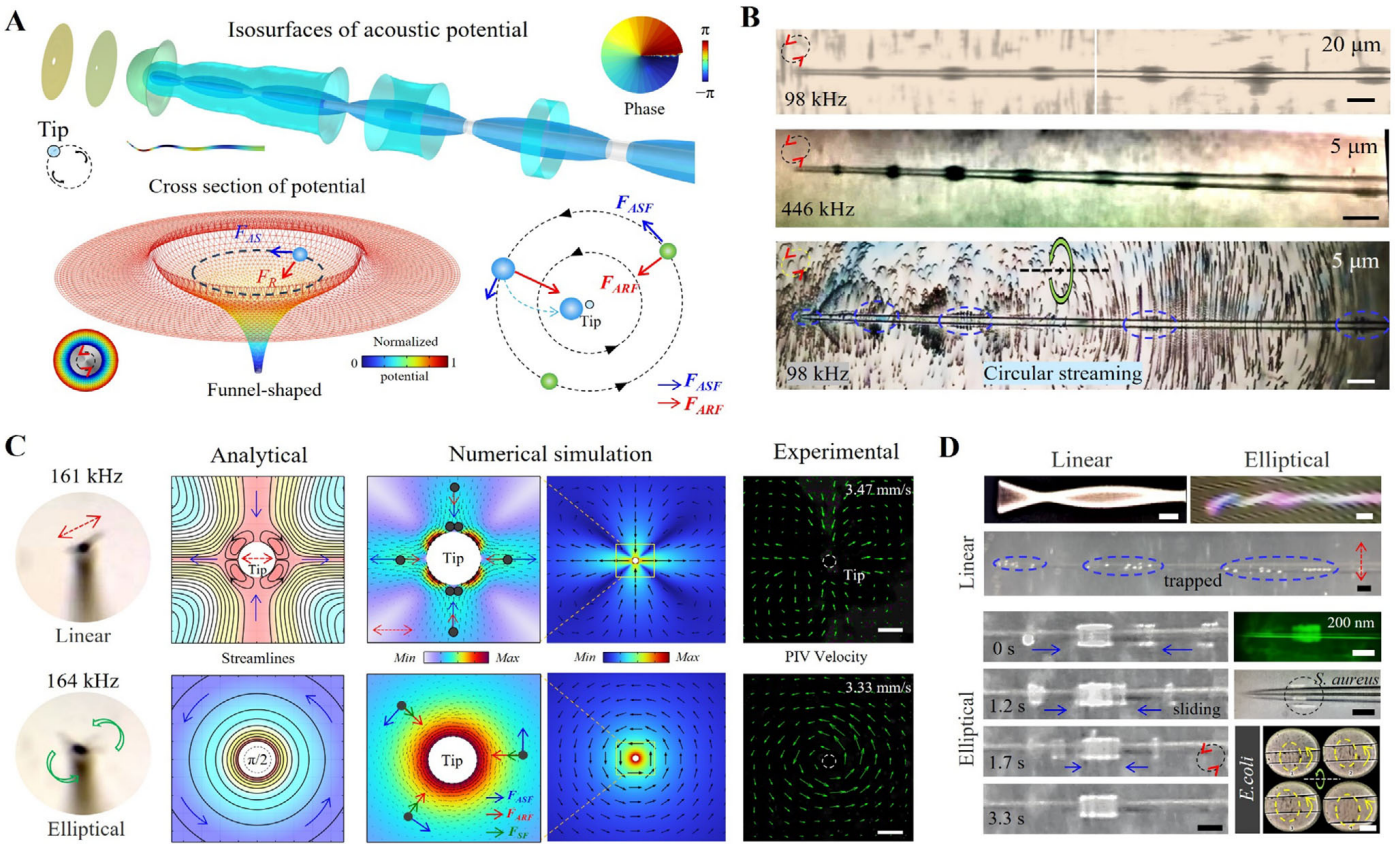
Physical mechanism of vibrating fiber‐tip and corresponding particle manipulation tests. A) Isosurfaces of the Gor'kov acoustic potential along the fiber‐tip axis, and the “funnel‐shaped” acoustic potential well in the cross‐section. The combined effect of the acoustic radiation force (*F_ARF_*) and the acoustic streaming force (*F_ASF_*) governs the particle kinematics. B) 3D trapping properties of vibrating fiber‐tip. Particles are trapped in localized regions along the axial direction of the fiber‐tip, corresponding to areas with small values of the 3D acoustic potential wells shown in A. C) Theoretical calculations, numerical simulations, and experimental validations for linear and elliptically‐polarized modes of vibration. The velocity field, generated using MATLAB based on particle image velocimetry (PIV) data, is indicated by green arrows showing particle velocity and direction. Linear vibration produces the characteristic “four vortices,” while elliptical vibration results in circular vortices. Scale bar: 40 µm. D) Comparison of the trapping capacities between the linear and torsional vibration modes along the fiber‐tip axis. The torsional vibration mode demonstrates superior trapping ability, inducing rapid particle rotation and directing particles toward the center of the potential well. Notably, 200 nm PS particles, *S. aureus*, and *E. coli* were effectively trapped under torsional vibration. Scale bar: 100 µm.

Along the axial direction of the fiber, we have systematically analyzed the 3D acoustic field characteristics of the vibrating fiber‐tip for the first time under standard vibration modes using a comprehensive numerical model. **Figure** **3**A illustrates the isosurfaces of the Gor'kov acoustic potential along the fiber axis, where the minimal values of the potential correspond to the maximal values of the amplitude, indicating regions of the stronger acoustic radiation forces (ARF). The overall vibration characteristics of the fiber exhibit a torsional vibration mode. The phase diagram on the right confirms the fiber‐tip vibration behavior that carries orbital angular momentum (OAM). The AS pattern induced by this mode is consistent with the findings of ZhenYu Hong et al. ^[^ ^36^ ^],^ who demonstrated that OAM‐carrying vortex acoustic fields can induce fluid rotation around the axis, with the rotational speed increasing as the distance from the center decreases. It should be noted that the torsional vibration mode in this paper refers to the vibration of the entire fiber, while the elliptical vibration mode only refers to the vibration of the tip.

The acoustic potential energy distribution for a cross‐section is depicted in Figure 3A, illustrating a “funnel‐shaped” acoustic potential well (presented in logarithmic scale due to the significant energy gradient). This trap structure is consistent across cross‐sections perpendicular to the fiber‐tip, and similar structures are observed in other vibration modes (linear and other elliptical vibrations are detailed in Figure S2, Supporting Information). Within the “funnel” trap, particles are influenced by both ARF and acoustic streaming forces (ASF): larger particles gradually converge toward the center, while smaller particles follow stable circular trajectories in specific orbits. Near the center of the “funnel” trap, the fluid velocity gradient increases sharply, forming a tornado‐like “low‐pressure zone.” The pronounced pressure difference (i.e., shear force (SF)) in this region may result in the particles being drawn into the center of the vortex, significantly altering their trajectories. Therefore, the behavior of the particles results from the combined effects of ARF, ASF, and SF.

As shown in Figure 3B, particles with diameters of 20 and 5 µm are observed to be trapped in localized regions along the axial direction of the fiber‐tip, respectively. These regions correspond to the regions of local minima of the 3D acoustic potential well, as predicted in Figure 3A. At lower vibration frequencies, 5 µm particles demonstrated varying degrees of circular motion around the fiber‐tip axis. As the ARF is inversely proportional to the acoustic wavelength, increasing the vibrational frequency can achieve stable capture even for small particles of 5 µm. It is noteworthy that exciting higher‐order vibration modes of the fiber‐tip requires a significant increase in input energy (from 10 to 20 Vpp) or modifications to the fiber‐tip's structural parameters (such as reducing the tip diameter to 10 µm) to modify its mass and stiffness properties. The difference between the particle trapping mechanisms in linear and elliptical vibrational modes at the fiber tip is investigated by a combination of analytical calculations, numerical simulations, and experimental validation (Figure 3C). The streaming pattern around the vibrating cylinder was calculated in the context of the small amplitude approximation and boundary layer theory (see Supporting Information for details). In the linear mode of vibration, the fluid direction is significantly deflected by 90°. Only a few particles are precariously trapped in the orthogonal direction of vibration, and the particles can easily escape along the vibration direction. In the elliptical vibration mode, the ASF is continuously orthogonal to the direction of the ARF, and the particles are stably pulled toward the vortex center. As shown in Figure 3D, the advantages of torsional vibrational modes in particle manipulation and enrichment are further demonstrated, showing superior performance compared to linear vibrational modes. This is attributed to the fact that the former effectively binds particles, causing them to rotate at high speeds and aggregate toward the center of the “funnel” trap (especially the tip region). This region becomes a pivotal site for particle enrichment and cell/bacteria lysis due to the high flow rate and concentrated forces. In addition, the ability of the torsional vibration mode to trap 200 nm polystyrene particles, *S. aureus*, and *E. coli* was further demonstrated (see Video S3, Supporting Information for details).

### 2.3 Lysis Performance and Enhanced Raman Spectra

#### 2.3.1 Lysis Performance

**Figure 4:**
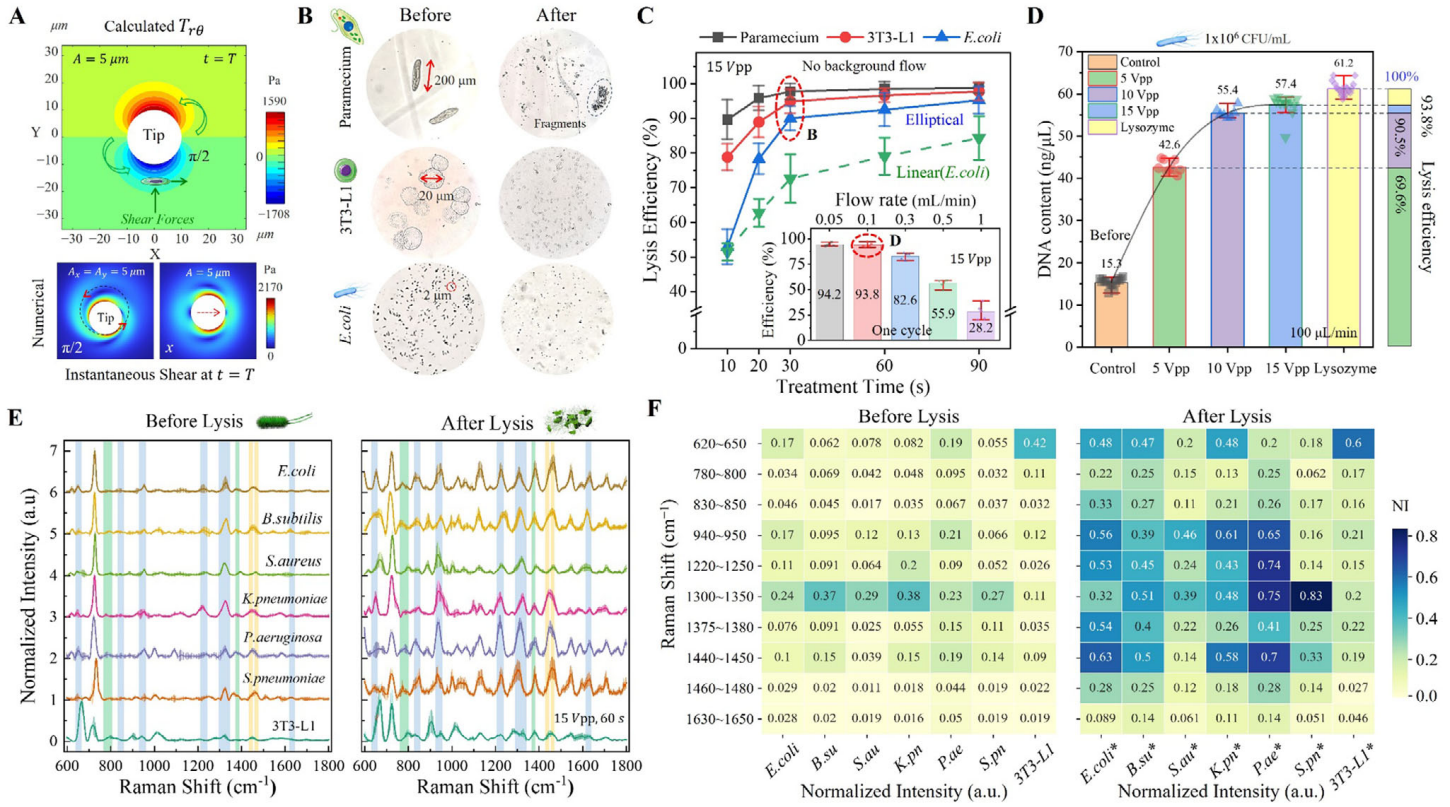
Lysis performance and enhanced Raman spectra. A) Instantaneous shear forces at *t* = *T* from calculations (small amplitude assumption, MATLAB) and numerical simulations (*f* = 50 kHz). B) Changes in cell and bacterial morphology before and after lysis with the torsional vibration mode. C) The effect of lysis time and background flow rate on lysis efficiency. D) Post‐lysis DNA content of *E. coli* compared to the lysozyme method. E) Averaged Raman spectra (mean ± standard deviation) of the seven bacterial species before and after lysis, with highlighted regions indicating significantly altered characteristic peaks. F) Heatmap visualizing significant Raman feature changes before and after lysis, clearly displaying variations in normalized intensity (NI) values through color gradients.

The rapid velocity and the highly concentrated force at the vibration center create a high‐intensity stress environment that cells cannot withstand, particularly the shear stress, leading to cell rupture. The instantaneous shear distribution at *t* = *T* from calculations and numerical simulations is illustrated in **Figure** **4**A (see SI for derivation). The circular‐polarized vibration generates a rotational shear distribution with a vortex‐like flow, while the *x*‐direction vibration produces a linear, wake‐like shear pattern aligned with the *x*‐axis. Therefore, the *x*‐direction mode promotes directional transport of particles along the axis of vibration, while the circular mode is more effective for creating rotational or trapping flows. This phenomenon is further supported by Figure S3 and Video S2 (Supporting Information). Video S2 (Supporting Information) offers both simulations and experimental results, demonstrating the vibrations and the corresponding acoustic streaming induced at the tip of the fiber.

To validate the broad applicability of the acoustofluidic lysis component, biological samples of varying scales were selected for testing: Paramecium (single‐cell model), Mouse embryonic fibroblasts (3T3‐L1, an animal cell model), and *E. coli* (bacterial model). Figure 4B shows the changes in cell and bacterial morphology following 30 s of treatment with the torsional vibration mode, demonstrating that the lyser is effective in lysing samples of different sizes (Video S4, Supporting Information). Due to the microfluidic environment, the large surface area enables rapid heat dissipation (Figure S4, Supporting Information), ensuring that temperature variations in the lyser do not significantly impact sample quality. Furthermore, the impact of lysis time and background flow rate on lysis efficiency was examined (Figure 4C). There are significant differences in the lysis characteristics of samples at varied sizes. Large cells, such as Paramecium and 3T3‐L1, demonstrate notable stress differences between the two sides of the cell membrane under shear conditions, leading to their rapid cleavage (lysis efficiency exceeding 90% within 30 s). In contrast, small bacteria (e.g., *E. coli*) have higher mechanical strength and can distribute shear forces more uniformly due to their smaller size, making them more difficult to lyse compared to larger cells. However, they are incapable of withstanding the sustained action of multiple stresses (e.g., SF, ARF, and ASF), requiring extended treatment times of 60 s or more to achieve higher lysis efficiency. In addition, the torsional vibration mode exhibits better lytic performance than the linear vibration mode due to its ability to continuously direct particles to regions of high intensity stress concentration. A plot of flow rate versus lysis efficiency (inset of Figure 4C) demonstrated that lysis efficiency is high when the flow rate is controlled below 100 µL min^−1^ and decreases significantly when the flow rate exceeds this threshold.

The evaluation methods for lysis efficiency varied depending on the sample type. Microscopic counting (Analyze Particles function in ImageJ) was employed for Paramecium and 3T3‐L1 cells (Figure S5, Supporting Information), while the lysozyme method was used as a benchmark for *E. coli*, given its widely recognized effectiveness in lysing both Gram‐positive and Gram‐negative bacteria, including *E. coli* strains. Additionally, the post‐lysis DNA content of *E. coli* was measured using ultra‐micro spectrophotometry and compared with the lysozyme method (Figure 4D). The results indicate that the lysis efficiency is lower at low voltages, primarily due to the stress intensity (e.g., SF, ARF, and ASF) in the central region being directly regulated by the applied voltage. When the voltage is increased to 10 Vpp or higher, the cracking efficiency increases to over 90%. Therefore, increasing the applied voltage and reducing the flow rate effectively were both effective in improving lysis performance. For valuable or rare samples, it is recommended to lower the flow rate or increase the number of processing cycles to maximize lysis efficiency.

#### 2.3.2 Enhanced Raman Spectra

The characteristic expression of intracellular macromolecules (nucleic acids, proteins, lipids, etc.) in Raman spectra was significantly enhanced after the treatment of six bacterial species (*E. coli*, *B. subtilis*, *S. aureus*, *K. pneumoniae*, *P. aeruginosa*, *S. pneumoniae*) and one mammalian cell line (3T3‐L1) with our acoustofluidic lysis technique. The averaged Raman spectra (mean ± standard deviation) of the seven species, measured before and after lysis, are presented in Figure 4E. The regions of significant spectral changes are highlighted with colored shading: green shading corresponds to nucleic acid‐related peaks, blue shading indicates protein vibrational peaks, and gold shading highlights mixed peaks of proteins and lipids. Notably, the post‐lysis Raman spectra exhibit a pronounced increase in both peak intensity and number, particularly in the high wavenumber region, directly reflecting the release of key intracellular components such as nucleic acids and proteins. More details of the changes in Raman spectra before and after lysis are given in Figures S6–S8 (Supporting Information).

To visualize the significant changes in spectral features, the relative intensities of the highlighted spectral regions were calculated and displayed as heatmaps in Figure 4F, with the corresponding Raman peak assignments provided in Table S1 (Supporting Information). The presentation of normalized intensity (NI) values and the color gradient comparison in the heatmap demonstrates the advantages of the acoustofluidic lysis technique. These advantages include low spectral noise, high signal quality, and information richness compared to untreated samples. This improved approach is particularly powerful for detecting biological systems with complex envelope structures, such as bacteria, offering enhanced feature recognition and signal amplification. It also provides a new technological tool for constructing a comprehensive cellular Raman “fingerprint” database.

### 2.4 Deep Learning for Bacterial Classification Based on Enhanced Raman Spectra

After lysis treatment, the information content and interspecies differences in bacterial spectra are significantly enhanced, which brings new challenges for further analysis and identification of spectra. In recent years, ML and DL techniques have shown exceptional performance in processing high‐dimensional spectral datasets.^[^ ^37^ ^]^ Among them, convolutional neural networks (CNNs),^[^ ^38^, ^39^, ^40^ ^]^ which originated in the field of image categorization, have performed particularly well. However, transferring these advanced CNN techniques, such as residual networks (ResNet) ^[^ ^41^ ^]^ to 1D spectral data is still in its infancy. Chi‐Sing Ho et al.^[^ ^42^ ^]^ They were the first to apply a 1D‐CNN based on the ResNet architecture to successfully identify 30 common pathogens, demonstrating the great potential of ResNet in spectral analysis.

**Figure 5:**
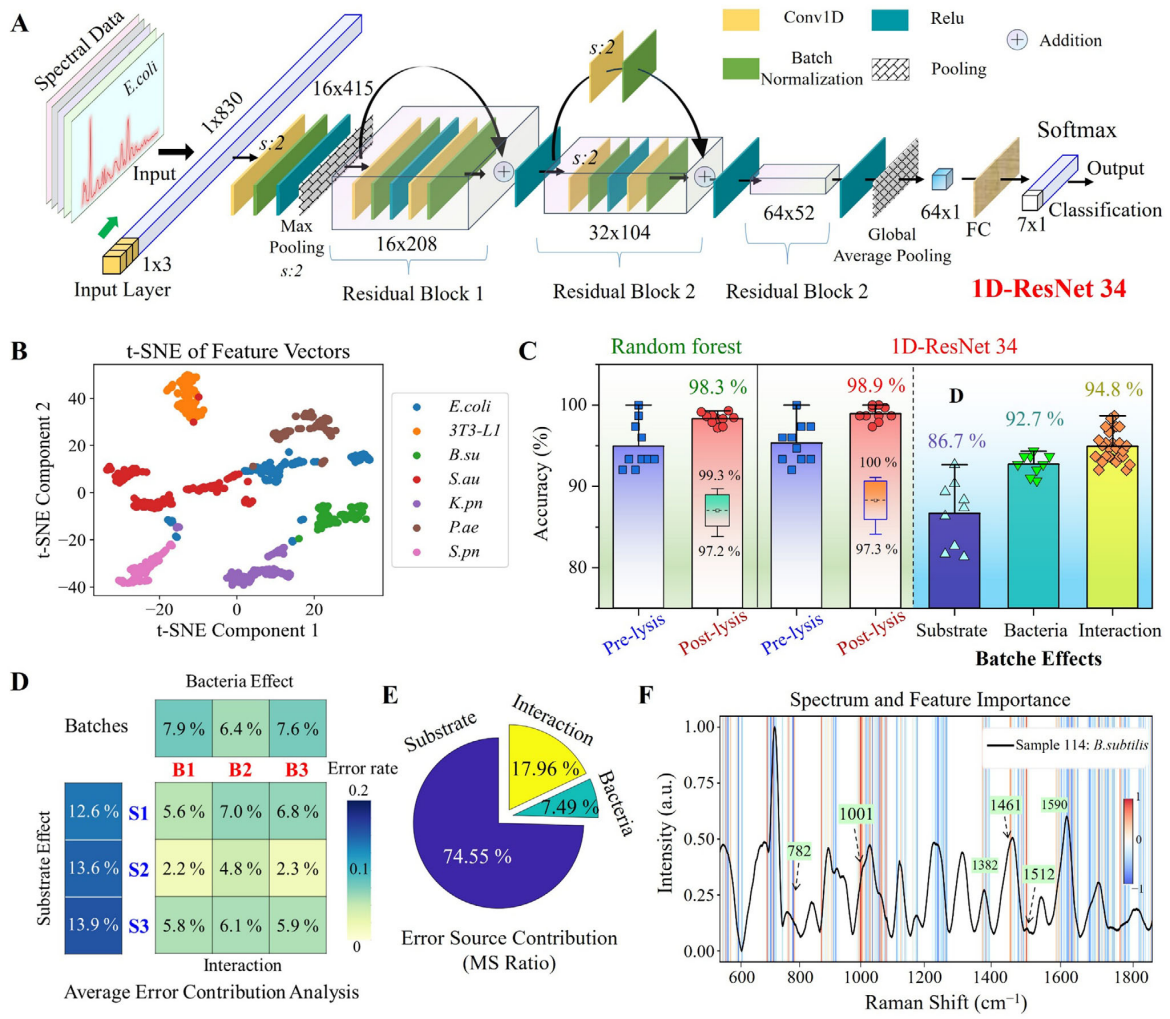
Deep learning for bacterial classification using enhanced Raman spectra. A) Schematic architecture of the 1D convolutional neural network (1D‐CNN) architecture based on ResNet. B) The 2‐component t‐distributed stochastic neighbor embedding (t‐SNE) projection with perplexity = 30, showing separation across all seven bacterial classes. C) Comparison of classification accuracy before and after lysis using the 1D‐ResNet and random forests models, and evaluation of batch effects on model generalization. D) Average error contribution heatmap across substrate batches (S1–S3), bacterial batches (B1–B3), and their combinations (3 × 3 matrix). E) Error source contribution pie chart, showing the proportions of variance explained by substrate effect (74.55%), bacterial batch effect (7.49%), and substrate–bacteria interaction (17.96%). F) Heatmap of the model's SHAP values, illustrating feature importance during the decision‐making process for a specific prediction (*Bacillus subtilis*).

Based on this, we designed a 1D‐ResNet architecture specifically for spectral data analysis (**Figure** **5**A). The model begins with an initial convolutional layer (kernel size of 3, with 16 filters) to extract low‐level features, using the ReLU activation function to enhance nonlinear mapping capabilities. Subsequently, two residual modules are employed to progressively deepen the network, with the number of filters increasing from 16 to 64. The residual connections prevent information loss and accelerate feature learning. Finally, the model uses global average pooling and a softmax layer for classification. This architecture not only enhances classification performance but also maintains training efficiency and improves the ability to discern subtle differences in spectral data.

We constructed two datasets for comprehensive model evaluation (Figure S9, Supporting Information). Principal Component Analysis (PCA) was initially employed for dataset 1 structure visualization (Figure S10, Supporting Information), yet significant overlap among several bacterial types indicates limitations in fully distinguishing all classes. Compared to PCA, the 2‐component t‐distributed stochastic neighbor embedding (t‐SNE) shown in Figure 5B provides a more distinct separation, revealing clearer differences between bacterial classes. We further conducted comparative experiments before and after lysis using a Random Forest classifier and the proposed 1D‐ResNet model (Figure 5C). Notably, prior to lysis, the spectral quality was relatively low, resulting in considerable fluctuations in classification accuracy, particularly for small sample sizes (Figure S11, Supporting Information). This trend was observed in both the Random Forest and ResNet models, indicating that classification performance in pre‐lysis samples was more susceptible to environmental noise, signal intensity variations, baseline fluctuations, and other confounding factors. After lysis, both models maintained high classification stability and accuracy across iterations (all above 97%). This significant performance improvement is attributed to the contributions of the acoustofluidic lysis technique in the bacteria preprocessing stage, which effectively enhanced data quality and feature representation. These results underscore the critical role of the lysis procedure in enhancing spectral consistency and improving classification robustness. Consequently, our integrated approach achieved high classification accuracies (98.3% with a random forest and 98.9% with the 1D‐ResNet model), demonstrating that the combination of acoustofluidic lysis with Raman spectroscopy and deep learning substantially enhances both data quality and overall model performance. To further evaluate the impact of batch effects on model generalization, we introduced three distinct nested cross‐validation (CV) frameworks, each designed to isolate and assess a particular source of error—substrate batch, bacterial batch, and their interaction (see *Training Details*). A summary of the test accuracies for each held‐out fold, along with the mean error heatmap across the three schemes, is presented in Figure S12 (Supporting Information) and Figure 5D. The color scales represent the error rates, clearly highlighting that substrate batch variability is the predominant source of error. Notably, even when tested on completely unseen substrate–bacteria combinations (Scheme S3, Supporting Information), the 1D‐ResNet model still achieves high accuracy (94.8%), indicating strong generalizability despite batch variability. To quantitatively assess the relative contribution of each factor, we further calculated the Error Source Contribution (MS Ratio) (Figure 5E). Specifically, substrate variability contributed 74.55% of the total error variance, confirming it as the dominant error source. The relatively low contribution of 7.49% from bacterial batches suggests that biological variability across batches is modest (e.g., better control of culture conditions), even though the average per‐batch error slightly exceeded the interaction effect (Figure 5D). The interaction term, despite only moderate average combination errors, contributed ≈18% of the total variance. This reflects the nonlinear synergistic effects between substrate properties and bacterial phenotypes, revealing the system's complexity. See Error‐Source Analysis and Implication in Supporting Information for more Details. A summary of the error effect types, underlying mechanisms, and their contributions to data variability is provided in **Table** **1**. By following a tiered mitigation strategy: first minimizing systemic substrate‐induced errors, then reducing biological batch variability, and finally accounting for synergistic substrate–bacteria interactions, we anticipate achieving a substantial reduction in total diagnostic error, thereby enhancing the robustness and reproducibility of SERS‐based bacterial classification for real‐world applications.

**Table 1:** Summary of Error Effect Types, Their Mechanisms, and Contributions to Data Variability.

Effect Type	Underlying Mechanism	Impact Scope	Pattern of Data Variation	MS Contribution Sensitivity
Substrate Effect	Systematic Global Shift	Global	Cross‐batch systematic deviations	High (broad coverage)
Bacterial Effect	Class‐Specific Local Shift	Local	Consistent deviations within species	Moderate (narrow coverage)
Interaction Effect	Nonlinear Synergistic Shift	Combination‐specific	Consistent deviations within species	Low overall, but with sharp peaks

To elucidate the decision‐making process of the deep learning model in classification tasks, SHAP (SHapley Additive Explanations) values were employed to quantify feature importance. Figure 5F presents a SHAP values heatmap for *Bacillus subtilis*, highlighting the key spectral features that contributed to the classification task. Specifically, the Raman peak at 1001 cm⁻¹ was assigned the highest weight, corresponding to the pyridine ring vibrational mode of calcium dipicolinate (CaDPA) or dipicolinic acid (DPA).^[^ ^43^, ^44^ ^]^ This is a critical biomarker of *B. subtilis* spores, underscoring its significant role in distinguishing this species.^[^ ^45^ ^]^ Additionally, minor peaks at 1512 cm⁻¹ (C═C stretching) and 1517 cm⁻¹ (C─C stretching) were also given notable weights, indicating the presence of carotenoids, particularly β‐carotene.^[^ ^46^ ^]^ These are essential components in *B. subtilis* metabolism and antioxidant processes.^[^ ^47^ ^]^ Furthermore, the Raman features at 782 cm⁻¹ (pyridine ring vibration), 1382 cm⁻¹ (C─H and CH₃ bending), 1461 cm⁻¹ (CH₂/CH₃ bending), and 1590 cm⁻¹ (C═C stretching) may indicate the presence of nucleic acids, lipids, peptidoglycans, and carotenoids in *B. subtilis*.^[^ ^48^ ^]^ Figure S13 (Supporting Information) provides SHAP value heatmaps for other bacterial species as a reference. These key spectral features not only reflect the biological characteristics of various bacteria but also inform the model's classification decisions, thereby significantly enhancing classification performance.

### 2.5 Clinical Application Potential

**Figure 6:**
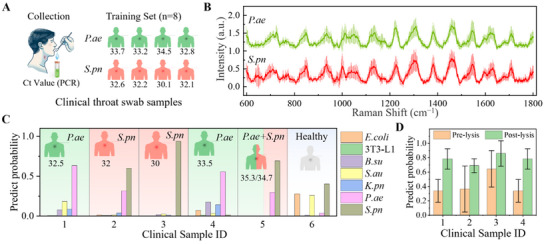
Clinical Application Potential. A) Collection of clinical throat swab samples for the additional training set, with samples subjected to the same processing conditions (15 Vpp, 60 s) and 3–5 valid Raman spectra collected per sample. B) Averaged Raman spectra (mean ± standard deviation) of *P. aeruginosa* (*P.ae*) and *S. pneumoniae* (*S.pn*) from patient samples. C) Predicted probability bar chart for six clinical samples, illustrating the potential of the method for predicting clinical samples with high accuracy. D) Comparison of predicted average probabilities for pre‐lysis and post‐lysis samples, demonstrating higher prediction accuracy and robustness after lysis.

To enhance the model's performance and clinical applicability, we incorporated additional clinical samples directly collected from patient throat swabs without prior isolation or culturing. *P. aeruginosa* (*P.ae*) and *S. pneumoniae* (*S.pn*) bacterial samples collected from patients were used to augment the training set. Each sample was subjected to the same processing conditions (15 Vpp, 60 s), and 3–5 valid Raman spectra were collected per sample. These data were used to train the model for more robust classification of bacterial species (**Figure** **6**A). The averaged Raman spectra (mean ± standard deviation) of *P.ae* and *S.pn* from patient samples are presented in Figure 6B.

The predicted probability bar chart for the six clinical samples confirms the potential of our method for clinical applications (Figure 6C). High prediction probabilities were observed for both *P. aeruginosa* and *S. pneumoniae*, indicating the model's ability to accurately classify clinical samples, even in the absence of traditional culturing methods. Furthermore, a comparison between pre‐lysis and post‐lysis samples (Figure 6D) revealed that pre‐lysis samples exhibited higher variability and less reliable predictions, whereas post‐lysis samples demonstrated significantly improved consistency and robustness. Additionally, a detailed prediction analysis for Clinical Sample No. 1 is shown in Figure S14 (Supporting Information). The prediction probability distribution for five individual Raman spectra obtained before lysis (Figure S14A, Supporting Information) versus after lysis (Figure S14B, Supporting Information) clearly illustrates that the lysis procedure enhances prediction accuracy and robustness. These findings underscore the critical role of the lysis step in improving the reliability of bacterial identification from clinical samples. Compared to conventional bacterial identification methods (Table S2, Supporting Information), our acoustofluidic‐SERS‐ML approach achieves rapid, high‐sensitivity detection while minimizing preprocessing requirements, demonstrating strong potential for direct clinical applications.

## 3 Conclusion

In this study, we demonstrate for the first time a novel interdisciplinary approach that integrates acoustofluidic lysis, Raman spectroscopy, and deep learning for rapid, label‐free, and accurate bacterial classification. We propose an acoustofluidic lysis mechanism based on a vibrating fiber‐tip that effectively exposes intracellular biomolecules such as nucleic acids, proteins, and lipids. This process significantly enhances the expression of features in bacterial Raman spectra, improving both spectral resolution and information richness. By combining these enhanced spectra with the 1D‐ResNet model, our approach achieves excellent classification accuracy and robustness, with an average accuracy of 98.9%, surpassing traditional classifiers like random forests. Even when tested on completely unseen substrate‐bacteria combinations, a high accuracy (94.8%) was still achieved, demonstrating strong generalisability. More importantly, the supplemental clinical experiments demonstrate that our method can accurately identify bacterial species without the need for pre‐culture and colony isolation. As our clinical database continues to expand, our approach has the potential to deliver rapid, point‐of‐care diagnostics, particularly in scenarios where timely decision‐making is critical.

## 4 Experimental Section

### Device Fabrication

The fiber‐tip was fabricated from a bare quartz fiber with a diameter of 0.5 mm, and the tip diameter ranged between 10 and 20 µm. This fiber was bonded to a bimorph piezoelectric transducer (PZT5H, XHXDZ, China), with the tip extending ≈3 cm beyond the transducer. The transducer, with dimensions 7.0 × 8.0 × 0.2 mm, was encapsulated in a custom 3D‐printed housing and connected to a function generator (FeelElec, China). The fiber‐tip was precisely positioned inside a glass capillary with an outer diameter of 2.4 mm, an inner diameter of 1 mm, and a length of 5 cm, ensuring coaxial alignment with the extension channel of the 3D‐printed housing (Figure 1A). Finally, the capillary channel was connected to a peristaltic pump (LEFOO, China) and a three‐way valve via Teflon tubing with an inner diameter of 2 mm.

### Numerical Analysis

The nonlinear acoustic streaming, instantaneous shear forces, and acoustic potential well distribution induced by the vibrating fiber‐tip were investigated using FEM software. First, a 2D model was used to simulate the tip vibration, employing pressure acoustics, solid mechanics, and laminar flow modules to solve for the nonlinear acoustic streaming induced by the tip vibration (Figure S1 and Table S3, Supporting Information). This process can be approximated using the time‐averaged Navier‐Stokes equation:

(1) $$\frac{\partial u^{\left(2\right)}}{\partial t}+\left(u^{\left(1\right)}\cdot \nabla \right)u^{\left(1\right)}=-\frac{1}{\rho_{0}}\nabla p^{\left(2\right)}+v\nabla^{2}u^{\left(2\right)}$$

Here, 〈*u* ^(1)^ · ∇*u* ^(1)^〉 is the time‐averaged convective term of the first‐order velocity field, *u* ^(2)^ is the second‐order velocity field, 〈*p* ^(2)^〉 is the second‐order steady‐state pressure, and *v* is the kinematic viscosity of the fluid.

Next, moving mesh techniques were applied to simulate the fluid‐structure interaction between the solid mechanics and laminar flow modules, analyzing the instantaneous flow velocity and shear forces generated by different tip vibration modes (*x* (*t*) = *A_x_* sin (ω*t*), *y* (*t*) = *A_y_* sin (ω*t* − φ)). The radial shear force T_*r*θ_ is caused by the radial gradient of the tangential velocity *u*_θ_(*r*,θ, *t*), while the tangential shear force T_θ*r*_ arises from the tangential gradient of the radial velocity *u_r_* (*r*,θ, *t*):

(2) $$\;T_{r\theta }=\mu \frac{\partial u_{\theta }\left(t\right)}{\partial r},T_{\theta r}=\mu \frac{1}{r}\frac{\partial \left(ru_{r}\left(t\right)\right)}{\partial r}$$

where μ is the dynamic viscosity of the fluid.

Finally, a 3D model was constructed, using the acoustics and solid mechanics modules to systematically solve the vibration modes and acoustic pressure field distribution within the capillary tube (Figures S15 and S16, Supporting Information). The Gor'kov potential *U* was then obtained from the acoustic pressure field:

(3) $$U=\frac{4\pi a^{3}}{3}2\kappa_{0}p_{0}^{2}\left[\frac{1}{2\rho_{0}c_{0}^{2}}f_{1}\langle p^{2}\rangle -\frac{3\rho_{0}}{4}f_{2}\langle v^{2}\rangle \right]$$

From this, the acoustic radiation force *F_ARF_* =   − ∇*U* was calculated,^[^ ^49^ ^]^ where ρ_0_, ρ_*p*_, *c*_0_, and *c_p_* are the densities and sound speeds of the fluid and particle, respectively. 〈*p* ^2^〉 and 〈*v* ^2^〉 are respectively the mean‐square fluctuations of the acoustic pressure and velocity. The monopole and dipole scattering coefficients *f*_1_ and *f*_2_ are expressed as:

(4) $$f_{1}=1-\frac{c_{0}^{2}\rho_{0}}{c_{p}^{2}\rho_{p}},f_{2}=\frac{2\left(\rho_{p}-\rho_{0}\right)}{2\rho_{p}+\rho_{0}}$$

The acoustic streaming drag force was calculated using the Stokes force formula:

(5) $$F_{ASF}=6\pi \mu a\left(u_{0}-u_{p}\right)$$

where *u*_0_ and *u_p_* are the instantaneous velocities of the fluid and particle, respectively.

### Batch Preparation

SERS‐substrate batches: Three independently fabricated silver nanoparticles (AgNPs) SERS substrate batches (S1, S2, S3) were prepared to account for batch‐to‐batch variability. The batch differences were characterized by SEM and UV–Vis spectroscopy (Figure S17, Supporting Information). Bacterial batches: Three independent bacterial culture batches (B1, B2, B3) were grown from cryopreserved standard strains (China Medical Culture Collection Center, CMCC) using plate streaking (Figure S18, Supporting Information).

### Raman Measurements

Bacteria were isolated from agar plates prior to each experiment. A 1 mg biomass from a single colony was suspended in 100 µL of phosphate‐buffered saline (PBS), achieving a concentration of ≈1 × 10^8^ CFU mL^−1^, as determined by Nanodrop ultra‐micro spectrophotometry (Thermo Fisher, USA). Five technical replicates of lysate (15 Vpp, 60 s acoustofluidic lysis) were prepared, then each lysate (15 µL) was split into three ≈5 µL aliquots and deposited onto three independently fabricated silver‐nanoparticle SERS substrates (S1, S2, S3). After air‐drying, ten SERS spectra were acquired per substrate replicate, yielding 50 spectra per species per substrate batch (Figure S19, Supporting Information). SERS measurements were conducted using a Raman spectrometer (B&W Tek, USA) with a laser wavelength of 785 nm and intensity of ≈10 mW, exposing the sample for 3 s and accumulating 3 scans.

### Spectra Pre‐Processing

Spectra were pre‐processed in three steps: 1) smoothing, 2) baseline correction, and 3) normalization, all done using the Python 3.12 programming language. Smoothing was performed with the Savitzky−Golay method using an 11‐pixel window and polynomial order of 3. Baseline correction was performed by adaptive iterative reweighting penalised least squares (airPLS) (Figure S20, Supporting Information). Spectra were scaled using MinMaxsScaler to have a minimum value of 0 and a maximum value of 1.

### Datasets Structure

Dataset 1 comprises 700 pre‐lysis spectra and 2100 post‐lysis spectra, collected from seven species before and after acoustofluidic lysis (15 Vpp, 60 s) using a single substrate–bacteria combination (S1–B1). This dataset was primarily used to compare the classification performance before and after lysis using the 1D‐ResNet and random forest models. Dataset 2 consists of 2700 post‐lysis spectra collected from nine fully independent substrate–bacteria combinations (3 substrate batches × 3 bacterial batches), specifically designed to assess batch‐level variability effects, including substrate batch effects, bacterial batch effects, and their interaction (Figure S12, Supporting Information).

### Training Details

Dataset 1 was partitioned into 10 equal‐sized subsets. For each outer fold, one subset was used exclusively for testing, while the remaining nine were used in a 9 fold inner cross‐validation for Bayesian hyperparameter optimization.

Dataset 2: Implemented strict nested cross‐validation (NCV) with three independent validation schemes (Figure S21, Supporting Information). Scheme S1 (Supporting Information) (Substrate‐out CV): Isolates substrate effects under fixed bacterial conditions. Utilizes a 3 fold outer CV (excluding substrate batches) to reserve one substrate group (S1‐S3) for testing, paired with a 2 fold inner CV for hyperparameter tuning on the remaining data. Scheme S2 (Supporting Information) (Bacteria‐out CV): Evaluates bacterial effects under fixed substrate conditions. Mirrors Scheme S1 (Supporting Information) with a 3 fold outer CV (excluding bacterial batches, B1‐B3) and a 2 fold inner CV to ensure unbiased estimation of bacterial‐specific performance. Scheme S3 (Supporting Information) (Strict Combo‐out CV): Tests generalization to unseen substrate‐bacteria combinations. Employs a 9 fold outer CV (excluding unique substrate‐bacteria pairs) and an 8‐fold inner CV, enforcing strict separation of training, validation, and test data to simulate real‐world deployment on novel combinations.

Bayesian Optimization: Three key hyperparameters were optimized over the following ranges: Initial Learn Rate: 1 × 10⁻⁴ to 1 × 10⁻^2^. MiniBatch Size: 16 to 128. Max Epochs: 10 to 80. For each outer fold, Bayesian optimization runs for eight iterations, using the “expected‐improvement‐plus” acquisition function to balance exploration and exploitation (Figure S22, Supporting Information). The loss function utilized was categorical cross‐entropy, allowing for effective multi‐class classification. An early stopping mechanism was activated when validation loss ceased to improve, ensuring optimal model performance (Figure S23, Supporting Information).

For additional details, please refer to the Model Validation section in the Supporting Information.

### Clinical Sample Processing and Raman Measurement

To enhance the model's performance and clinical applicability, additional clinical samples, directly collected from patient throat swabs Nanjing Children's Hospital in 2023, were incorporated without prior isolation or culturing. For the clinical samples with Ct values ranging from 30 to 35, which correspond to bacterial concentrations of ≈10⁴–10^2^ CFU mL^−1^, the bacteria were further concentrated by centrifugation. The specific protocol involved centrifuging at 8000 rpm for 3 min, removing the supernatant, and concentrating the sample to ≈20 µL. The vibrating fiber‐tip lysis system was then employed, which takes advantage of capillary action for processing small volumes of the concentrated samples. Each sample was subjected to the same processing conditions (15 Vpp, 60 s), and 3–5 valid Raman spectra were collected for each sample (Figure S24, Supporting Information).

### Ethical Approval

All procedures involving human materials in this study were approved by the ethical Review Committee of Jiangsu Provincial Center for Disease Control and Prevention (JSJK2025‐B005‐01). Consent was obtained from all participants prior to the study.

## Conflict of Interest

The authors declare no conflict of interest.

## Supporting information



Supporting Information

Supporting Information

Supplemental Video 1

Supplemental Video 2

Supplemental Video 3

Supplemental Video 4

## Data Availability

The data that support the findings of this study are available from the corresponding author upon reasonable request.
