# Supplementary material for: Deep Learning‐Assisted Rapid Bacterial Classification Based on Raman Spectroscopy of Bacteria Lysed by Acoustically Driven Fiber‐Tip Vibration
Source: Adv Sci (Weinh). 2025 Jul 8;12(32):e07724. doi: 10.1002/advs.202507724 (PMC12407356; doi:10.1002/advs.202507724)
Supplement: Supplementary file 1 — Supporting Information [file ADVS-12-e07724-s003.docx]

Supplementary Code S1

Main Cross-Validation Procedure for Scheme 3 (Combo-out CV)

This code implements the main cross-validation pipeline under Scheme 3 (strict combo-out cross-validation).
In this strategy, each substrate–bacteria combination is systematically held out in the outer loop for testing, while the remaining combinations are used in the inner loop for hyperparameter tuning via Bayesian optimization.

Specifically:

- **Outer Loop**: Leave-one-combination-out cross-validation. Each of the 9 substrate–bacteria combinations (e.g., S1-B1, S1-B2, ..., S3-B3) is used once as an independent test set.
- **Inner Loop**: Leave-one-out cross-validation among the remaining 8 combinations, using Bayesian optimization to tune hyperparameters.
- **Hyperparameter Search Space**:
  - Initial Learning Rate: [10^−4^,10^−1^] (log-transformed)
  - Mini-Batch Size: [16,128] (integer)
  - Max Epochs: [10,80] (integer)
- **Optimization**: 8 rounds of Bayesian optimization (MaxObjectiveEvaluations = 8) are performed in each inner loop to select the best hyperparameter set.

After tuning, the optimized model is trained on the entire inner-loop training set and evaluated on the outer-loop held-out fold.

***Scheme 3_main***

%% ---------- Scheme 3 (Visualization and Hyperparameter Recording) ----------

fprintf('\n===== Scheme 3: Outer CV Leave-One-Group-Out, Inner CV Leave-One-Out + Bayesian Optimization Visualization =====\n');

numFolds = numFolders;

outerCVResults = zeros(numFolds,1);

bestParamsAll = table('Size',[numFolds,4], ...

'VariableTypes',{'string','double','double','double'}, ...

'VariableNames',{'Fold','InitialLearnRate','MiniBatchSize','MaxEpochs'});

for i = 1:numFolds

fprintf('\n>>> Outer CV: Testing Folder %s <<<\n', validFieldNames{i});

testIdx = i;

trainIdx = setdiff(1:numFolders, testIdx);

% Extract training and testing data

[X_train, Y_train] = extractFeaturesLabels(data, validFieldNames(trainIdx));

[X_test, Y_test] = extractFeaturesLabels(data, validFieldNames(testIdx));

fprintf(' Training Samples = %d, Testing Samples = %d\n', size(X_train,1), size(X_test,1));

% Inner CV + Bayesian Optimization

optVars = [ ...

optimizableVariable('InitialLearnRate',[1e-4,1e-1],'Transform','log'), ...

optimizableVariable('MiniBatchSize',[16,128],'Type','integer'), ...

optimizableVariable('MaxEpochs',[10,80],'Type','integer')];

results = bayesopt(@(params) cvObjective_Scheme3(params, data, trainIdx), ...

optVars, ...

'Verbose',1, ...

'AcquisitionFunctionName','expected-improvement-plus', ...

'MaxObjectiveEvaluations',8, ...

'PlotFcn',{@plotObjective,@plotMinObjective,@plotConstraintModels}, ...

'UseParallel',false);

best = results.XAtMinObjective;

fprintf(' Best Hyperparameters: LR = %.4g, Batch Size = %d, Epochs = %d\n', ...

best.InitialLearnRate, best.MiniBatchSize, best.MaxEpochs);

% Train outer-loop model using optimal hyperparameters

hyper.InitialLearnRate = best.InitialLearnRate;

hyper.MiniBatchSize = best.MiniBatchSize;

hyper.MaxEpochs = best.MaxEpochs;

model_outer = trainResNet(X_train, Y_train, hyper);

outerAcc = predictAndVisualize(model_outer, X_test, Y_test);

fprintf(' Fold %d Test Accuracy = %.2f%%\n', i, outerAcc*100);

outerCVResults(i) = outerAcc;

% Record best hyperparameters for each fold

bestParamsAll.Fold{i} = validFieldNames{i};

bestParamsAll.InitialLearnRate(i) = best.InitialLearnRate;

bestParamsAll.MiniBatchSize(i) = best.MiniBatchSize;

bestParamsAll.MaxEpochs(i) = best.MaxEpochs;

end

% Save cross-validation results

save('cv_results_scheme3_Pro.mat','outerCVResults','bestParamsAll');

writetable(bestParamsAll,'Scheme3_BestHyperparams.csv');
