# Supplementary material for: Deep Learning‐Assisted Rapid Bacterial Classification Based on Raman Spectroscopy of Bacteria Lysed by Acoustically Driven Fiber‐Tip Vibration
Source: Adv Sci (Weinh). 2025 Jul 8;12(32):e07724. doi: 10.1002/advs.202507724 (PMC12407356; doi:10.1002/advs.202507724)
Supplement: Supplementary file 2 — Supporting Information [file ADVS-12-e07724-s002.docx]

Supporting Information

**Deep Learning-Assisted Rapid Bacterial Classification Based on Raman Spectroscopy of Bacteria Lysed by Acoustically Driven Fiber-tip Vibration**

*Yukai Liu, Miaomiao Ji, Xiao Ren, Zhenyong Dong, Tian Wen, Qingyue Dong, Ho-pui Ho, Lunbiao Cui*, Yanqing Lu*, Guanghui Wang**

Y. Liu, M. Ji, X. Ren, Z. Dong, Y. Lu, G. Wang

Key Laboratory of Intelligent Optical Sensing and Integration of the Ministry of Education

College of Engineering and Applied Sciences

Nanjing University

Nanjing, Jiangsu 210023, PR China

E-mail: [wangguanghui@nju.edu.cn](mailto:wangguanghui@nju.edu.cn) (Guanghui Wang), yqlu@nju.edu.cn (Yanqing Lu)

T. Wen, L. Cui

NHC Key laboratory of Enteric Pathogenic Microbiology

Jiangsu Provincial Medical Key Laboratory of Pathogenic Microbiology in Emerging Major Infectious Diseases

Jiangsu Provincial Center for Disease Control and Prevention

Nanjing, Jiangsu 210009, PR China

E-mail: [lbcui@jscdc.cn](mailto:lbcui@jscdc.cn) (Lunbiao Cui)

Q. Dong, H. Ho

Department of Biomedical Engineering

The Chinese University of Hong Kong

Shatin, Hong Kong SAR, 999077, China

**Frequency-dependent streaming at the capillary tip**

***Numerical Analysis***

In the viscous boundary layer, near the vibrating surface, fluid motion is influenced by viscosity, generating net flow due to asymmetric shear forces. This flow typically extends outward from the surface along the vibration plane. The process can be approximated using the time-averaged Navier-Stokes equation as follows:

$$\begin{aligned} \frac{\partial\boldsymbol{u}^{\left( 2 \right)}}{\partial t}+\left( \boldsymbol{u}^{\left( 1 \right)}\cdot\nabla\right)\boldsymbol{u}^{\left( 1 \right)}=-\frac{1}{\rho_{0}}\nabla p^{\left( 2 \right)}+v\nabla^{2}\boldsymbol{u}^{\left( 2 \right)}\#\left( 1 \right) \end{aligned}$$

Given the focus on second-order steady-state acoustic streaming, the time derivative term vanishes, and the time-averaged equation simplifies to:

$$\begin{aligned} \left\langle\boldsymbol{u}^{\left( 1 \right)}\cdot\nabla\boldsymbol{u}^{\left( 1 \right)} \right\rangle=-\frac{1}{\rho_{0}}\nabla\left\langle p^{\left( 2 \right)} \right\rangle+v\nabla^{2}\boldsymbol{u}^{\left( 2 \right)}\#\left( 2 \right) \end{aligned}$$

Here, $\left\langle\boldsymbol{u}^{\left( 1 \right)}\cdot\nabla\boldsymbol{u}^{\left( 1 \right)} \right\rangle$ represents the time-averaged convective term of the first-order velocity field, $\boldsymbol{u}^{\left( 2 \right)}$ is the second-order velocity field, $\left\langle p^{\left( 2 \right)} \right\rangle$ is the second-order steady pressure, and $v$ is the kinematic viscosity of the fluid.

***Analytic Calculation***

The net flow induced by nonlinear effects is referred to as steady acoustic streaming, which is particularly pronounced around vibrating bodies. The primary driving force of this streaming arises from the viscous shear forces that form a viscous boundary layer (Stokes layer) near the vibrating surface. For a cylindrical oscillating body, one can refer to the case of a small sphere oscillating in a viscous fluid.^[1]^

Consider a cylinder with radius $R_{0}$. In this two-dimensional problem, it is convenient to work in polar coordinates $\left( r,\theta\right)$, where $x=rcos\theta$ and $y=rsin\theta$. The velocity field is expressed as $\boldsymbol{u}=\boldsymbol{(}u_{r},u_{\theta}\boldsymbol{)}$, and we introduce the stream function $\psi$ such that:

$$\begin{aligned} u_{r}\boldsymbol{=}r^{-1}\frac{\partial\psi}{\partial\theta},u_{\theta}\boldsymbol{=-}\frac{\partial\psi}{\partial\theta}\#\left( 3 \right) \end{aligned}$$

In the first case, we consider linear vibrations occurring solely in one direction (e.g., along the *y*-axis) with an amplitude $A_{y}$. In this scenario, the streaming velocity potential function is defined as: ^[1-2]^

$$\begin{aligned} \psi=\frac{27}{40}\varepsilon^{2}\delta\left( \frac{1}{r^{2}}-1 \right) {sin}^{2}\theta cos\theta\#\left( 4 \right) \end{aligned}$$

Here, where $\delta=\left( 2v/\omega\right)^{1/2}$is the thickness of the oscillatory shear layer, or "Stokes layer", near the surface of the cylinder. $\varepsilon={A_{y}}/a$ is the ratio of the displacement amplitude $A_{y}$ to the radius of the cylinder.

In the second case, we consider simultaneous vibrations in both the *x*-axis and *y*-axis directions. If the vibrations are in phase, the effect is equivalent to the previous linear vibration case, where $A_{x}=A_{y}$ manifests as a single vibration along the line at $\varphi=\pi/4$ in the *x*-*y* plane, with an amplitude of $\sqrt{2}A_{x}$. For a cylinder with radius $R_{0}$ oscillating in the fluid, the motion can be expressed as:

$$\begin{aligned} x\left( t \right)=A_{x}\sin\left( \omega t \right), y\left( t \right)=A_{y}\sin\left( \omega t-\varphi\right)\#\left( 5 \right) \end{aligned}$$

Applying boundary layer theory, the velocity within the boundary layer rapidly decays, yielding:

$$\begin{aligned} u_{x}\left( r,t \right)=A_{x}\omega\cos\left( \omega t \right)\cdot e^{-\kappa\left( r-R_{0} \right)}\#\left( 6 \right) \end{aligned}$$

$$\begin{aligned} u_{y}\left( r,t \right)=-A_{y}\omega\cos\left( \omega t-\varphi\right)\cdot e^{-\kappa\left( r-R_{0} \right)}\#\left( 7 \right) \end{aligned}$$

Here, $\kappa=1/\delta$ is the decay coefficient within the Stokes boundary layer.

To simplify the analysis, consider a case where $A_{x}=A_{y}=A$ and $\varphi=\pi/2$ (indicating *y* lags *x* by $\pi/2$). In this scenario, the trajectory of the oscillation describes a counterclockwise circular motion. The mean velocity field induced by the viscous shear forces (i.e., the acoustic streaming velocity field) can be obtained through time averaging of the oscillatory velocity field. We focus on the nonlinear term in the velocity field, $\left\langle\boldsymbol{u}^{\left( 1 \right)}\cdot\nabla\boldsymbol{u}^{\left( 1 \right)} \right\rangle$. Under the assumptions of small amplitude and low Reynolds number within the boundary layer theory, the radial and tangential components of the second-order steady-state acoustic streaming are approximated as follows:

$$\begin{aligned} u_{r}^{\left( 2 \right)}=-\frac{A\omega^{2}}{2}\cdot\frac{R_{0}^{2}}{r^{3}}e^{-2\kappa\left( r-R_{0} \right)}\#\left( 8 \right) \end{aligned}$$

$$\begin{aligned} u_{\theta}^{\left( 2 \right)}=\frac{A\omega^{2}}{2}\cdot\frac{R_{0}}{r^{2}}e^{-2\kappa\left( r-R_{0} \right)}\#\left( 9 \right) \end{aligned}$$

***Primary Contributor to Acoustic Radiation Force***

The Gor'kov potential comprises contributions from both monopole (pressure) and dipole (velocity) components. Although one might intuitively expect particles to be trapped at nodes of minimal acoustic pressure (i.e., the equilibrium positions), in our system, the geometry of the narrow fiber-tip leads to a scenario in which acoustic energy is primarily conveyed through vibrational motion rather than static pressure. Consequently, the gradient of the velocity field dominates over that of the pressure field, making the dipole (velocity) term the primary contributor to the acoustic radiation force.

As a result, particles are effectively trapped at regions of maximum vibration (where the local fluctuations of the acoustic velocity are greatest) rather than at the pressure nodes. This phenomenon is further illustrated in Figure S15.

***Instantaneous Shear Forces***

The steady-state response of the fluid is closely related to nonlinear effects and acoustic streaming, while the instantaneous response is significantly influenced by transient flow induced by vibrations. Given that we are focused on the instantaneous shear forces caused by these vibrations, we can approximate using the first-order velocity gradient.

In polar coordinates, the instantaneous velocity field $\boldsymbol{u}\left( r,\theta,t \right)$ in the boundary layer near the cylinder at a distance $r$ from its center can be expressed as:

$$\begin{aligned} u_{r}\left( r,\theta,t \right)=u_{x}\left( r,t \right)\cos\theta+u_{y}\left( r,t \right)\sin\theta\#\left( 10 \right) \end{aligned}$$

$$\begin{aligned} u_{\theta}\left( r,\theta,t \right)=-u_{x}\left( r,t \right)\sin\theta+u_{y}\left( r,t \right)\cos\theta\#\left( 11 \right) \end{aligned}$$

The radial shear force $T_{r\theta}$ arises from the radial gradient of the tangential velocity $u_{\theta}\left( r,\theta,t \right)$, while the tangential shear force $T_{\theta r}$ is induced by the tangential gradient of the radial velocity $u_{r}\left( r,\theta,t \right)$. Thus, we have:

$$\begin{aligned} T_{r\theta}=\mu\frac{\partial u_{\theta}\left( t \right)}{\partial r}, T_{\theta r}=\mu\frac{1}{r}\frac{\partial\left( ru_{r}\left( t \right) \right)}{\partial r}\#\left( 12 \right) \end{aligned}$$

where $\mu$ is the dynamic viscosity of the fluid. Consequently, the expressions for the instantaneous shear forces in the radial and tangential directions become:

Considering the special case where $A_{x}=A_{y}=A$ and $\varphi=\pi/2$, the instantaneous shear forces simplify to:

$$\begin{aligned} T_{r\theta}\boldsymbol{=}\mu\kappa A\omega e^{-\kappa\left( r-R_{0} \right)}\sin\left( \omega t+\theta\right)\#\left( 13 \right) \end{aligned}$$

$$\begin{aligned} T_{\theta r}\boldsymbol{=-}\mu A\omega e^{-\kappa\left( r-R_{0} \right)}\sin\left( \omega t+\theta\right)\#\left( 14 \right) \end{aligned}$$

By calculating these expressions, we can approximate the instantaneous shear forces experienced by cells at any position $(r,\theta)$ near the vibrating surface. This understanding aids in elucidating the deformation and lysis of cells. The maximum instantaneous shear forces occur when $\sin\left( \omega t+\theta\right)=\pm1$, indicating that the cells experience their peak instantaneous shear forces at these moments. From the expressions, it is evident that the radial shear force is approximately $\kappa$ times the tangential shear force ($\kappa\approx3.96e^{5}$ when $f=50 kHz$). This indicates that the radial shear force plays a primary role in causing cell deformation and lysis.

Under the assumption of small amplitudes, the fluid motion is predominantly tangential (rotating around the cylinder). However, as the amplitude increases, nonlinear effects become significant, resulting in a pronounced increase in the tangential shear forces near the vibrating surface. This can be observed in Figure S3, which depicts transient vibrations at a specific moment, showing that due to viscous effects, the fluid generates a temporary "tail" near the oscillation trajectory. This phenomenon is especially pronounced for elliptical polarization of the vibration (see Supplementary Video S2).

**The Selection of the Acoustic Lysis Method**

The acoustic lysis methods encompass various approaches, including bulk acoustic waves (BAW) and surface acoustic waves (SAW). BAW methods primarily rely on strong cavitation effects, generating localized high-temperature and high-pressure regions that effectively disrupt cells.^[3]^ However, this process may cause thermal degradation and uneven lysis, potentially compromising sample integrity.^[4]^ SAW methods, on the other hand, utilize volumetric forces induced by high-frequency Rayleigh wave diffraction to drive fluid motion, producing relatively weak shear forces.^[5]^ While effective, SAW-based lysis often requires auxiliary structures such as microbeads^[6]^ or nanowires^[7]^ to enhance efficiency, which may introduce contaminants and complicate downstream analyses.

In contrast, our vibrating fiber tip approach offers several advantages. The localized, high-frequency vibration induces strong shear forces at the fiber tip, enabling efficient and controlled bacterial lysis without relying on external particles or excessive thermal effects. This approach ensures minimal sample contamination while preserving biomolecular integrity for downstream Raman spectral analysis.

**Variance Contribution**

Error Source Contribution: To further assess the relative contribution of each factor to the overall error variance, we calculated the Error Source Contribution (MS Ratio) (Figure 6E), which reflects the proportion of variance explained by each factor.

$$\begin{aligned} MS=\frac{1}{\text{N}}\sum_{i=1}^{N} \left( x_{i}-\bar{x} \right)^{2}\#\left( 15 \right) \end{aligned}$$

Where $N$ is the number of samples, $x_{i}$ are observed values, and $\bar{x}$ is the mean.

**SERS Measurements**

***Independent Spectral Acquisition Protocol*** (Figure S19A)

- **Lysis and sample preparation:** For each bacterial batch (e.g., B1: *E. coli*), five technical replicates of lysate were generated under uniform acoustofluidic conditions (15 Vpp, 60 seconds). Each lysate (15 μL) was divided into three aliquots (~5 μL) and deposited onto the three batches of AgNPs substrate (S1–S3) to ensure cross-batch validation.
- **SERS measurement:** After air-drying, 10 SERS spectra were collected per substrate replicate, yielding 50 spectra per bacterial species per substrate batch (5 lysate replicates × 10 spectra × 1 substrate batch). This protocol was repeated identically for all bacterial batches (B1–B3).

***Dataset Structure*** (Figure S19B)

- **Combinatorial design:** A 3×3 matrix of 9 substrate–bacteria combinations (S1-B1 to S3-B3) was constructed.
- **Data dimensions:** Each combination (e.g., S1-B1) comprises 50 spectra × 6 bacterial species, resulting in a cumulative dataset of 2,700 spectra (50 spectra × 6 species × 9 combinations).

**Model Validation**

***Validation Protocol Enhancement*** (Figure S21)

Implemented strict nested cross-validation (NCV) with three independent validation schemes:

- **Scheme 1 (Substrate-out CV)**: Isolates substrate effects under fixed bacterial conditions. Utilizes a 3-fold outer CV (excluding substrate batches) to reserve one substrate group (S1-S3) for testing, paired with a 2-fold inner CV for hyperparameter tuning on the remaining data.
- **Scheme 2 (Bacteria-out CV)**: Evaluates bacterial effects under fixed substrate conditions. Mirrors Scheme 1 with a 3-fold outer CV (excluding bacterial batches, B1-B3) and 2-fold inner CV to ensure unbiased estimation of bacterial-specific performance.
- **Scheme 3 (Strict Combo-out CV)**: Tests generalization to unseen substrate-bacteria combinations. Employs a 9-fold outer CV (excluding unique substrate-bacteria pairs) and 8-fold inner CV, enforcing strict separation of training, validation, and test data to simulate real-world deployment on novel combinations.

***Hyperparameter Optimization Process*** (Figure S22)

- **Outer Test Fold (e.g. S1–B1):** Under Scheme 3 (combo‑out CV), the S1–B1 combination is held out as the unseen outer test fold. Inner‑Loop CV: The remaining eight combinations serve as inner‑loop training/validation folds (leave‑one‑combo‑out), ensuring that no spectra from S1–B1 ever influence hyperparameter tuning.
- **Search Space:** Three key hyperparameters are optimized over the following ranges:

Initial Learn Rate: 1×10⁻⁴ to 1×10⁻². MiniBatch Size: 16 to 128. Max Epochs: 10 to 80.

- **Bayesian Optimization:** For each outer fold, Bayesian optimization runs for eight iterations, using the "expected‑improvement‑plus" acquisition function to balance exploration and exploitation.
- **Trace Monitoring:** At each iteration, we record the current validation error, the best‑so‑far minimum, and the estimated global minimum, and plot these as convergence traces (Figure S22C).
- **Independence of Test Data:** In all three schemes, outer‑loop test sets remain completely independent and are never used during any hyperparameter tuning step, ensuring an unbiased estimate of model generalization.

**Summary of Error‐Source Analysis and Implication**

To elucidate the dominant sources of classification error, we decomposed the total prediction variance into three orthogonal components:

1. **Substrate Effect** (Systematic Global Shift)

- **Description:** Variations in SERS‐substrate fabrication (e.g., nanoparticle size, interparticle spacing, surface roughness) introduce a reproducible, global bias across all bacterial spectra.
- **Mean Error:** 13.3 %
- **Variance Contribution (MS Ratio):** 74.55 %
- **Implication:** Because substrate‐to‐substrate variability overwhelmingly drives overall error, our foremost priority must be to tighten substrate fabrication tolerances—e.g. by standardizing nanoparticle deposition parameters or employing in‐line quality control—to suppress this global systematic bias.

1. **Bacterial** **Batch Effect** (Class‐Specific Local Shift)

- **Description:** Biological variability between independent cultures of the same species (e.g. growth phase, medium composition, cell density) produces modest, species‐specific spectral shifts.
- **Mean Error:** 7.3 %
- **Variance Contribution (MS Ratio):** 7.49 %
- **Implication:** Although the average per‐batch error slightly exceeds the interaction effect, its narrow MS contribution indicates that bacterial batch variability is relatively mild. Consistent culture conditions and standardized sampling can further reduce these local shifts.

1. **Interaction Effect** (Nonlinear Synergistic Shift)

- **Description:** Nonlinear interplay between substrate properties and bacterial phenotype yields combination‐specific spectral perturbations that cannot be ascribed to either factor alone (e.g. altered cell adhesion or “hot‐spot” coupling on rougher surfaces).
- **Mean Error:** 5.2 %
- **Variance Contribution (MS Ratio):** 17.96 %
- **Implication:** Even though individual combination errors are moderate, the interaction term accounts for nearly 18 % of the variance due to synergistic amplification of substrate‐ and biology‐driven effects. For example, when substrate surface‐roughness difference ΔRa > 0.2 μm, cell‐surface coupling undergoes an abrupt regime change, markedly affecting spectral features. As a result, process optimization must address not only each factor in isolation but also their coupled behavior, for instance by matching substrate roughness to specific bacterial targets or by developing adaptive calibration schemes.

**Practical Takeaway for Method Deployment:**

- **Priority 1:** Rigorously control and monitor substrate fabrication to minimize global biases.
- **Priority 2:** Standardize bacterial culture protocols to reduce class‐specific variability.
- **Priority 3:** Characterize and mitigate nonlinear substrate–bacteria interactions—e.g., by restricting substrate roughness within a calibrated window or by employing multivariate calibration that explicitly models interaction terms.

By following this tiered strategy: first eliminating systemic substrate errors, then attenuating biological batch shifts, and finally accounting for synergistic interactions. We anticipate the greatest reduction in total diagnostic error, thereby enhancing the robustness and reproducibility of SERS-based bacterial classification in real-world applications.


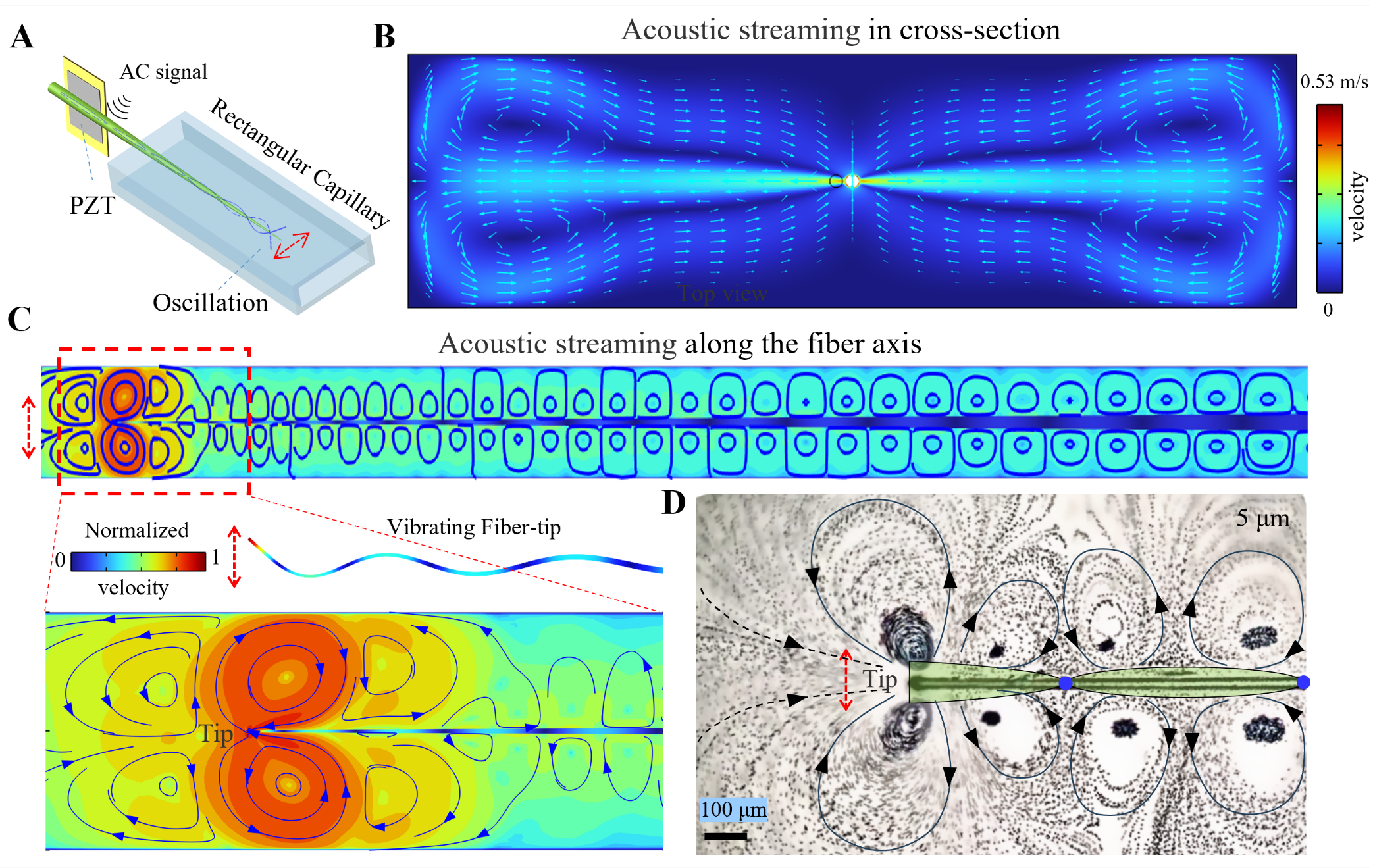


**Figure S1.** Acoustic streaming and trapping characteristics in a rectangular capillary under x-direction linear vibration mode. A) Vibrating fiber tip inside a rectangular capillary. B) Simulated acoustic streaming in the cross-section. The change in boundary conditions (rectangular capillary) flattens the typical "four-vortex" pattern. C) Acoustic streaming pattern along the axial direction of the capillary, showing multiple in-plane rotating vortices. D) Multiple in-plane rotating vortex patterns under linear vibration mode, obtained by time-lapse overlay. These vortices demonstrate the ability of the acoustic streaming to locally trap particles.


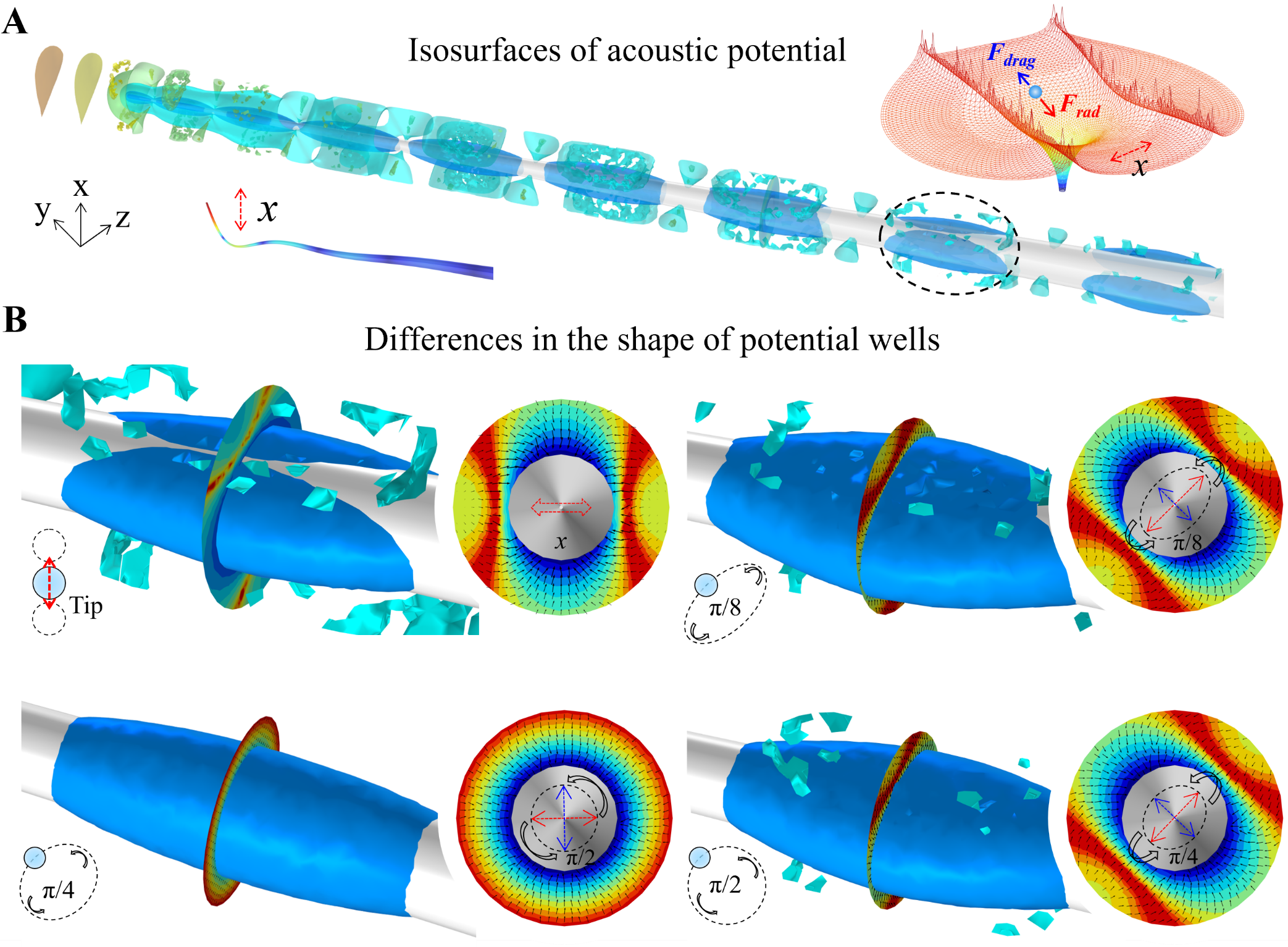


**Figure S2.** Differences in isosurfaces of acoustic potential distribution under different vibration modes induced by asymmetric vibrations. A) 3D acoustic potential well distribution during linear x-direction vibration, showing uneven distribution away from the tip. The inset displays the cross-sectional structure of the potential well, where a barrier forms in the vibration direction, while the gradient becomes shallower in the orthogonal direction. B) Shape of the acoustic potential wells under different vibration modes, with the inset showing cross-sectional potential and acoustic radiation force. As asymmetric vibration intensifies, the potential wells are progressively "flattened." Particles tend to accumulate along the short axis of the vibration trajectory (weaker vibration regions, as shown in the inset). The greater the long-to-short axis ratio [1, +∞], the more pronounced this effect becomes, especially farther from the tip.


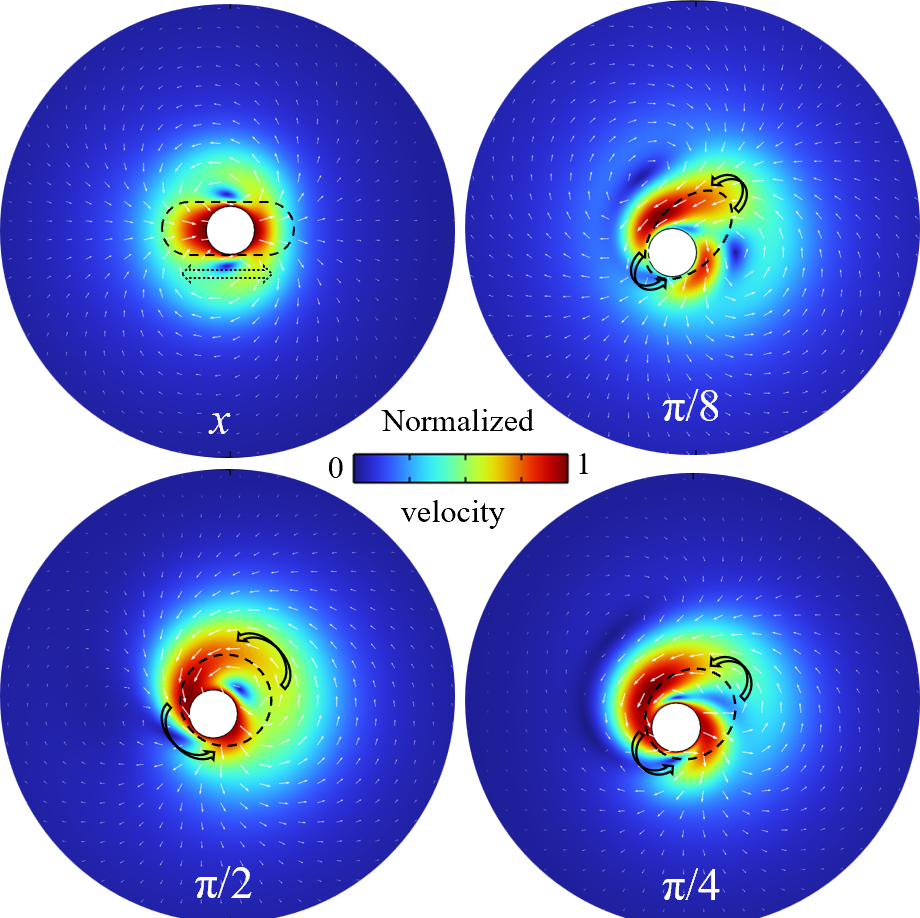


**Figure S3.** Simulated transient vibrations at a specific moment under different vibration modes. The rainbow color scale represents instantaneous fluid velocity, while arrows indicate the fluid flow direction. Due to viscous effects, a temporary "tail" forms near the oscillation trajectory.

***Temperature Characterization of the Acoustofluidic Lysis Device***

The analysis of the temperature variation of the acoustofluidic lysis device under normal operating conditions (46 kHz, 15 Vpp) to evaluate its potential impact on sample integrity. The experimental results are as follows:

We measured temperature changes in three distinct regions:

- Region 1: The liquid channel in direct contact with the transducer.
- Region 2: The vibrating fiber-tip region within the capillary.
- Region 3: The surrounding environmental temperature.

Due to the microfluidic environment, the large surface area facilitates rapid heat dissipation to the surroundings. Consequently, the maximum temperature increase observed was approximately 1°C, indicating that the device operates with minimal heating and does not significantly affect sample quality, including nucleic acids and proteins.


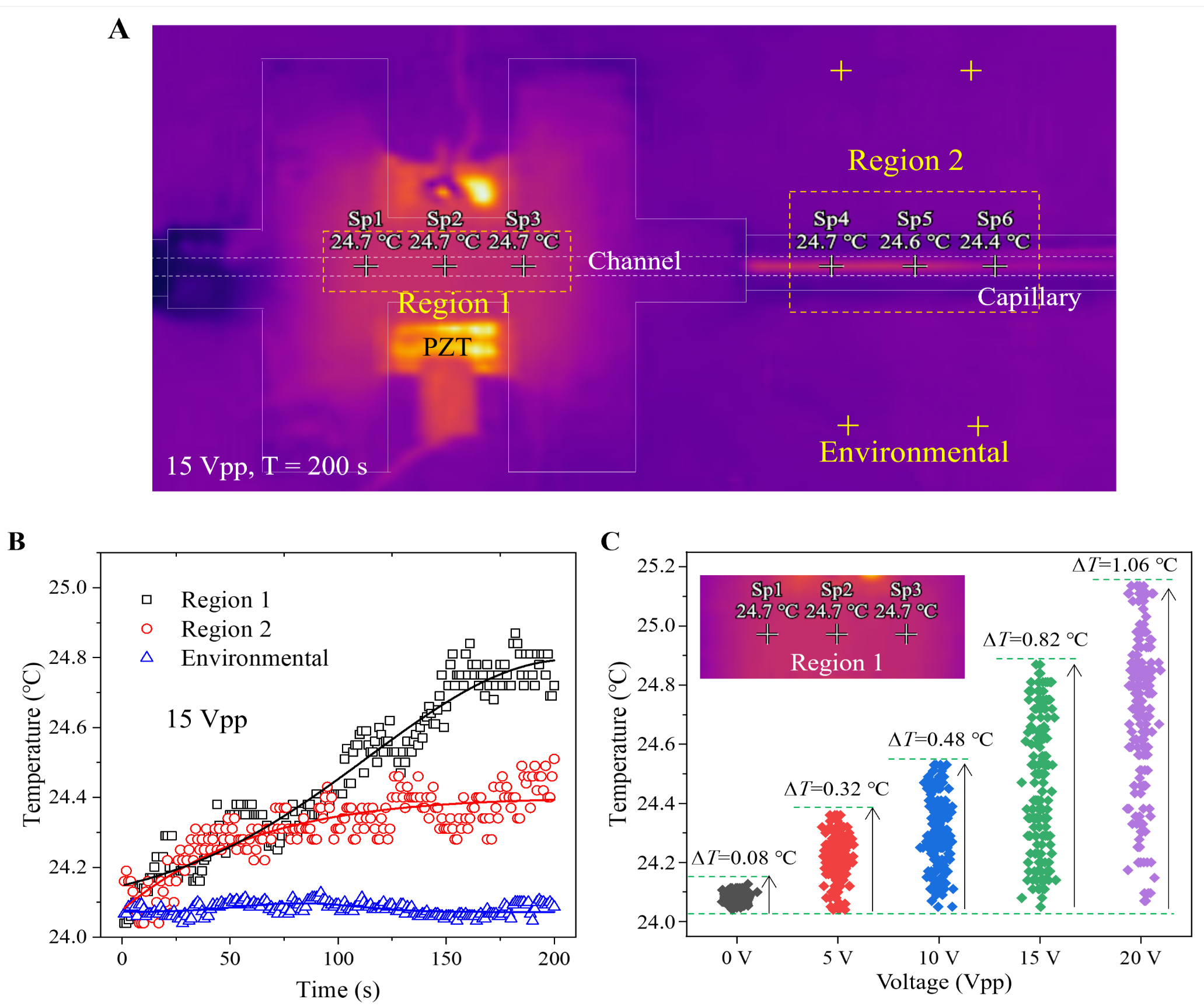


**Figure S4.** Temperature Characterization of the Acoustofluidic Lysis Device. A) Thermal imaging of the device with designated measurement regions. B) Temperature evolution over time in different regions. C) Temperature variation (*ΔT*) at different operating voltages.

***The Evaluation Methods for Lysis Efficiency***

The Analyze Particles function in ImageJ was used to quantify the total number of intact cells before lysis ($\text{N}_{\text{total}}$) and the remaining intact cells after lysis ($\text{N}_{\text{remaining}}$). The lysis efficiency (%) was calculated using the following formula:

$$\text{Lysis Efficiency(\%)=}\frac{\text{N}_{\text{total}}\text{-}\text{N}_{\text{remaining}}}{\text{N}_{\text{total}}}\text{×100\%}$$

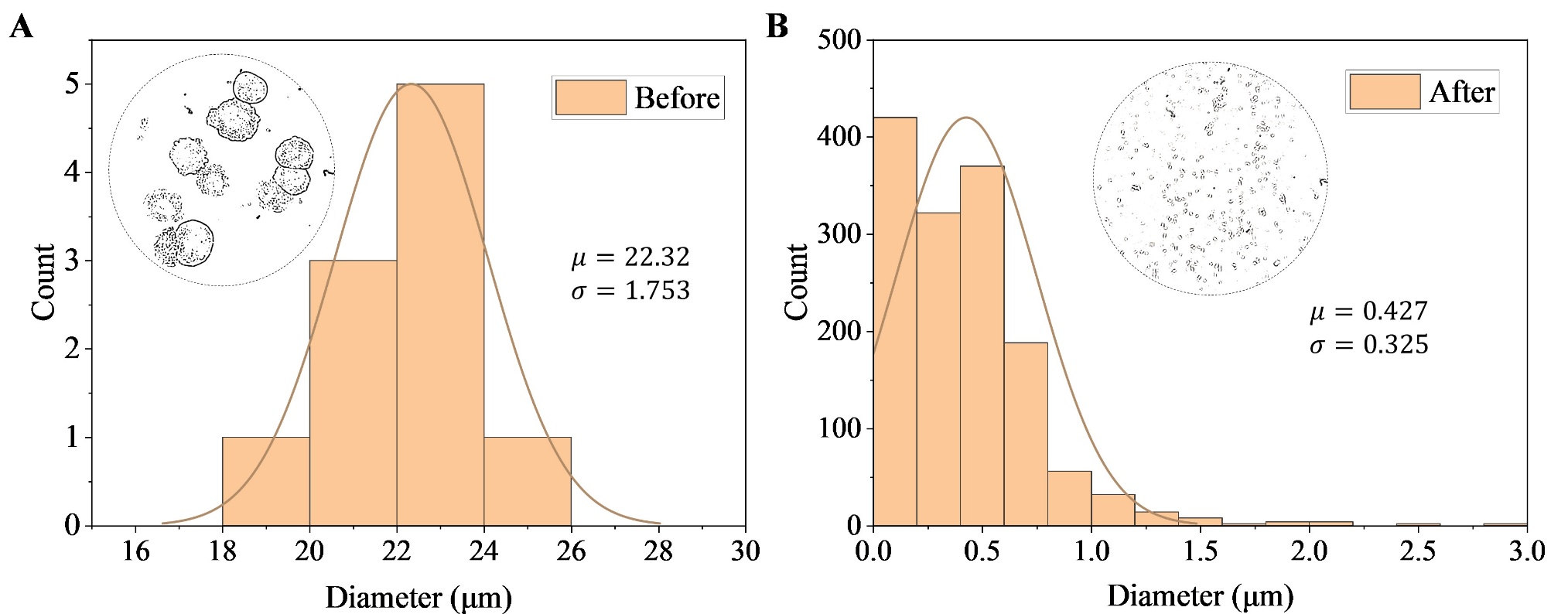


**Figure S5.** Particle size distribution histograms before A) and after B) lysis, analyzed using the Analyze Particles function in ImageJ. The insets display the corresponding microscopic images, while the mean (*μ*) and standard deviation (*σ*) of the particle diameters are annotated in each panel.


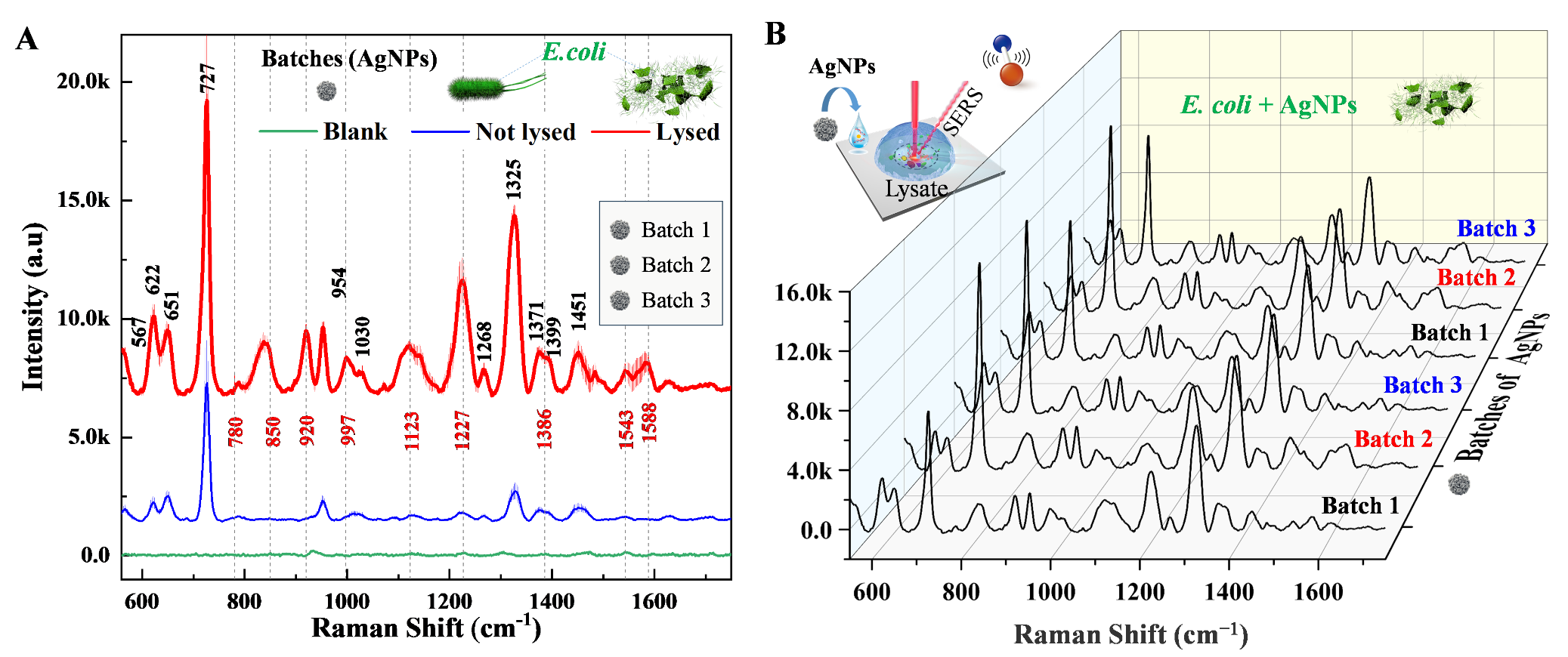


**Figure S6.** A) Averaged Raman spectra of *E. coli* samples before (blue) and after (red) lysis, measured on three different AgNPs batches, with 3–4 replicates per batch. B) Individual Raman spectra obtained from Batches 1, 2, and 3, presented as a 3D waterfall plot to assess batch-to-batch consistency in SERS signal acquisition.


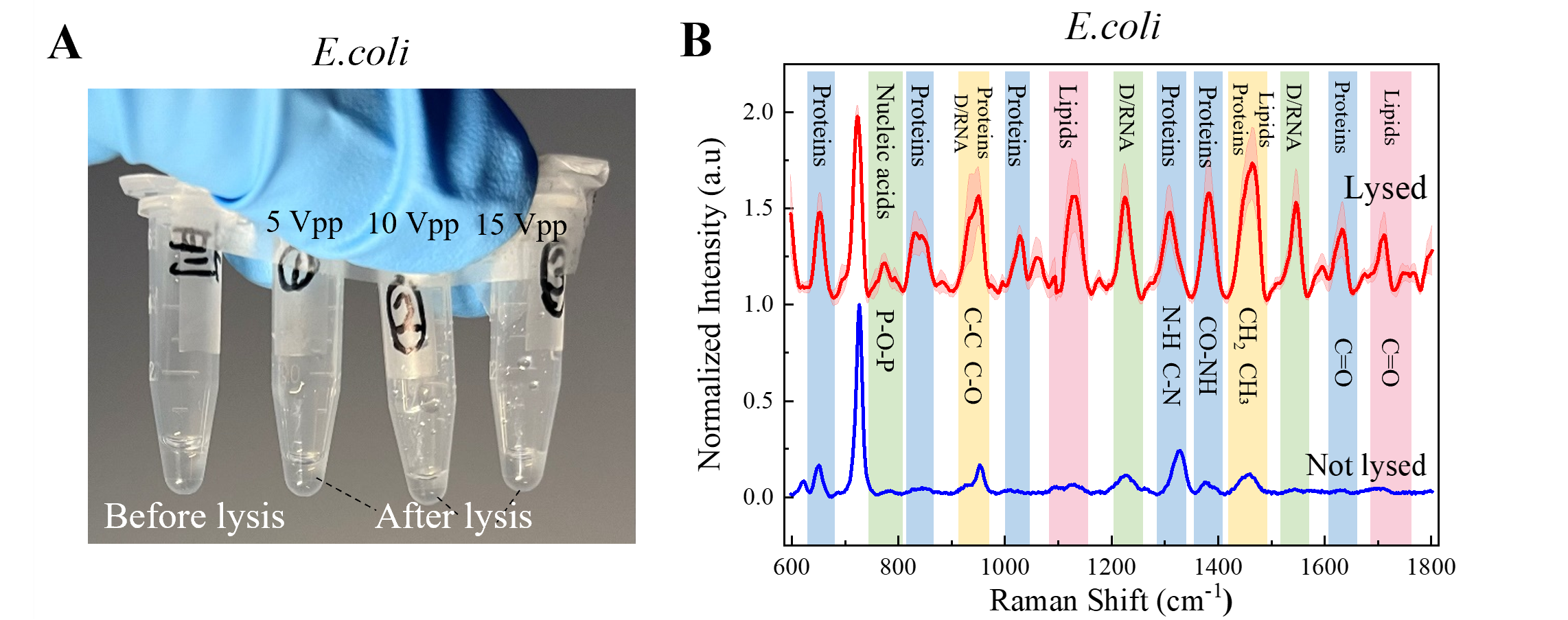


**Figure S7.** Changes in Raman spectra before and after lysis (*E. coli*). A) Untreated *E. coli* sample and collected samples treated at 5 Vpp, 10 Vpp and 15 Vpp. B) Significant Raman peak shifts before and after lysis, corresponding to key biomolecular structures. Prior to lysis, the spectra reflect intact cellular structures, with prominent peaks associated with proteins (e.g., Amide III at 1200–1280 cm⁻¹ and C-N stretch, N-H bend at 1300–1400 cm⁻¹), polysaccharides, and lipids (e.g., phospholipid P-O-P and CH₃). Post-lysis, the spectral changes indicate the release of intracellular components such as DNA/RNA (P-O-P backbone), proteins (C-C, C-H, C-N, CO-NH), and lipids (CH_2_, CH₃ and C=O stretching), signifying the breakdown of cellular membranes and the exposure of macromolecules.


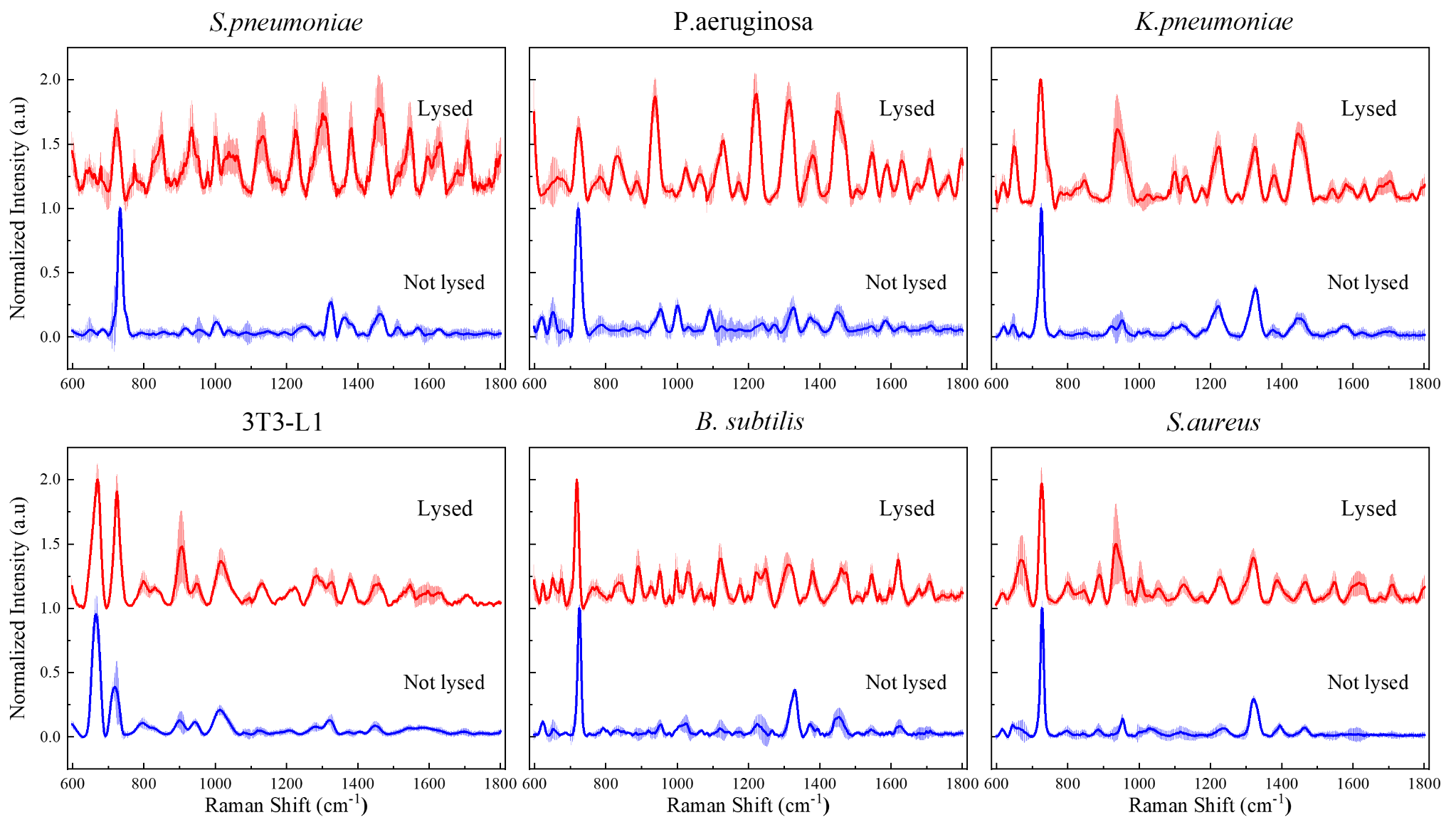


**Figure S8.** Comparison of Raman spectra before and after lysis for six additional samples. 3T3-L1 cells, representing animal cells, exhibited the smallest spectral variation, likely due to their thinner cell membrane, which does not hinder Raman spectroscopy from effectively detecting intracellular components. However, notable oscillations around the high wavenumber region near 1600 cm⁻¹ were observed, potentially reflecting protein release, conformational changes, and shifts in metabolite concentrations following lysis. Other bacterial samples showed varying degrees of spectral changes post-lysis, characterized by an increase in the number of Raman peaks, peak intensities, and shifts in peak positions.


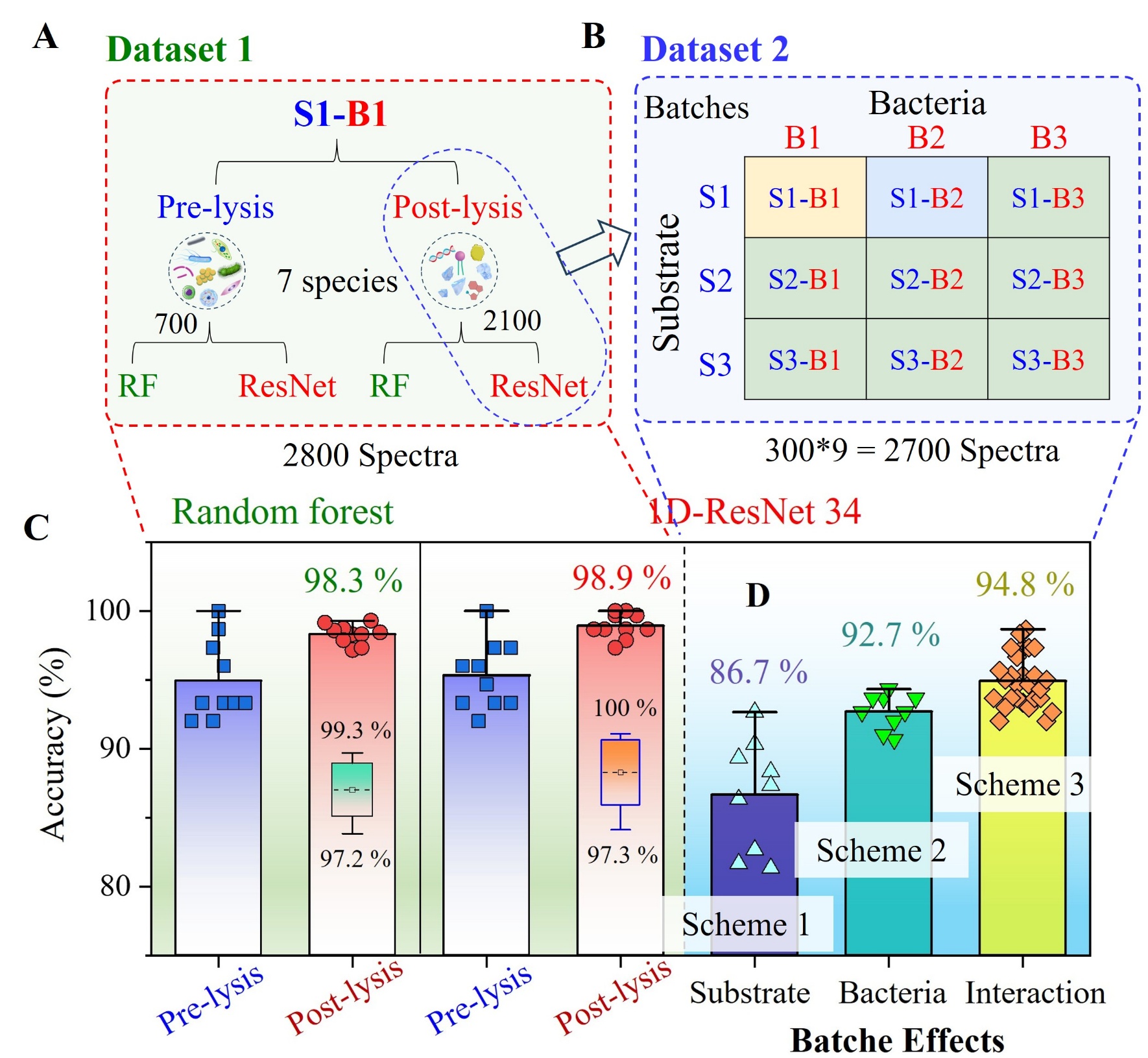


**Figure S9.** Dataset Overview and Model Validation Results. A) Dataset 1: Includes 2,800 spectra collected from 7 bacterial species before and after acoustic lysis using a single substrate–bacteria combination (S1–B1). This dataset was primarily used to compare the classification performance of the proposed 1D-ResNet model and a random forest (RF) model on both pre-lysis and post-lysis samples. B) Dataset 2: A newly constructed dataset comprising 2,700 post-lysis spectra collected from 9 fully independent substrate–bacteria combinations (3 substrates × 3 bacterial batches), used specifically for evaluating batch-level variability effects (substrate, bacterial batch, and their interaction). C) Classification accuracy on Dataset 1: Post-lysis data show significantly improved accuracy and lower variance compared to pre-lysis data, highlighting the effectiveness of acoustofluidic lysis. The 1D-ResNet model (98.9%) slightly outperforms the RF model (98.3%). D) Classification accuracy on Dataset 2 under different cross-validation schemes: Scheme 1 (Substrate-out CV), Scheme 2 (Bacteria-out CV), and Scheme 3 (Combo-out CV) demonstrate that batch effects significantly impact model performance. Notably, even when tested on completely unseen substrate–bacteria combinations (Scheme 3), the 1D-ResNet model still achieves high accuracy (94.8%), indicating strong generalizability despite batch variability.

**
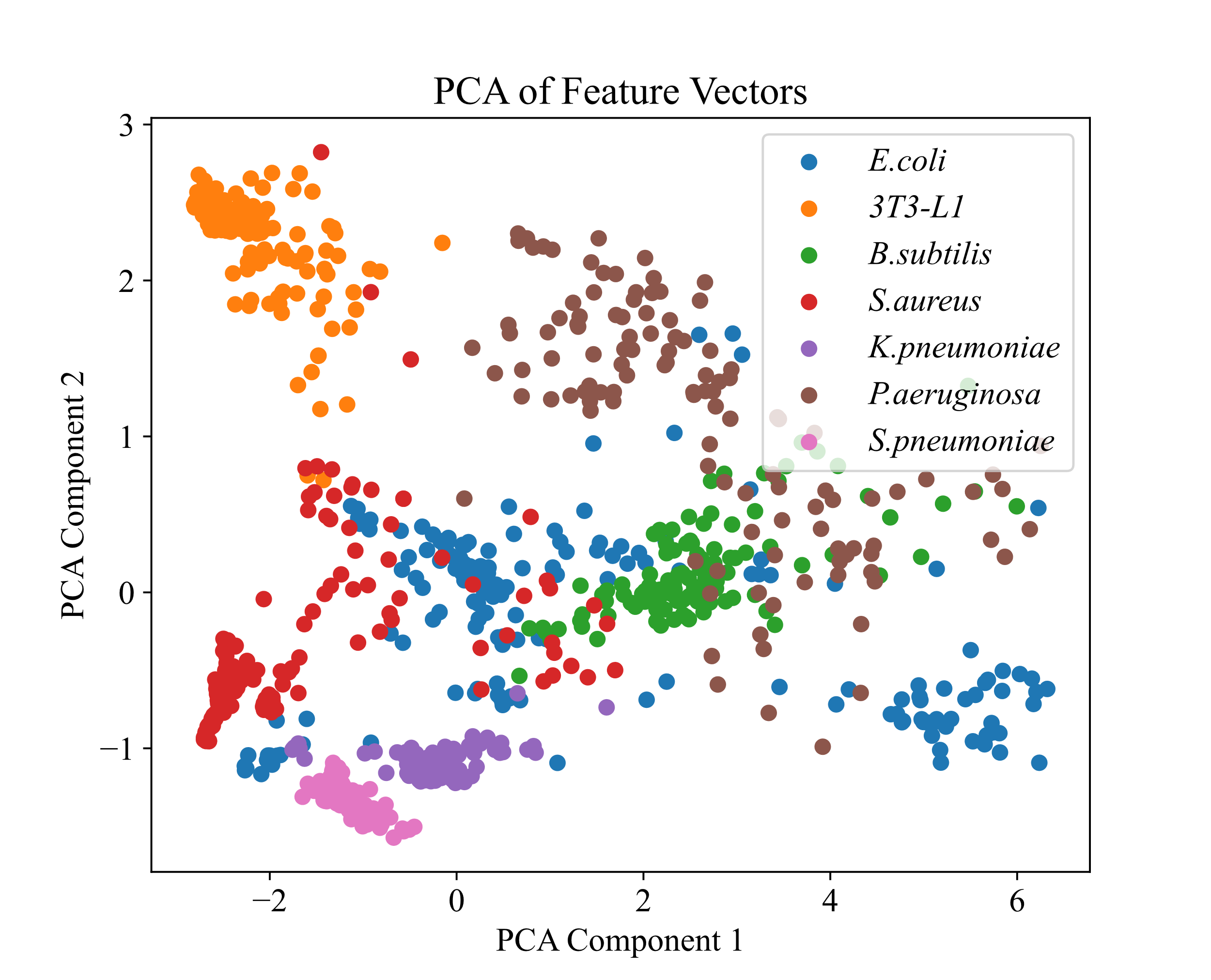
**

**Figure S10.** Principal Component Analysis (PCA) results for dataset structure visualization. PCA effectively highlights structural differences within the dataset, with clear distinction between the 3T3-L1 cell line and certain bacterial samples. However, the significant overlap observed among several bacterial types suggests that more advanced techniques, such as deeper machine learning models or alternative dimensionality reduction methods, may be required to achieve full separation and enhance classification accuracy across all classes.


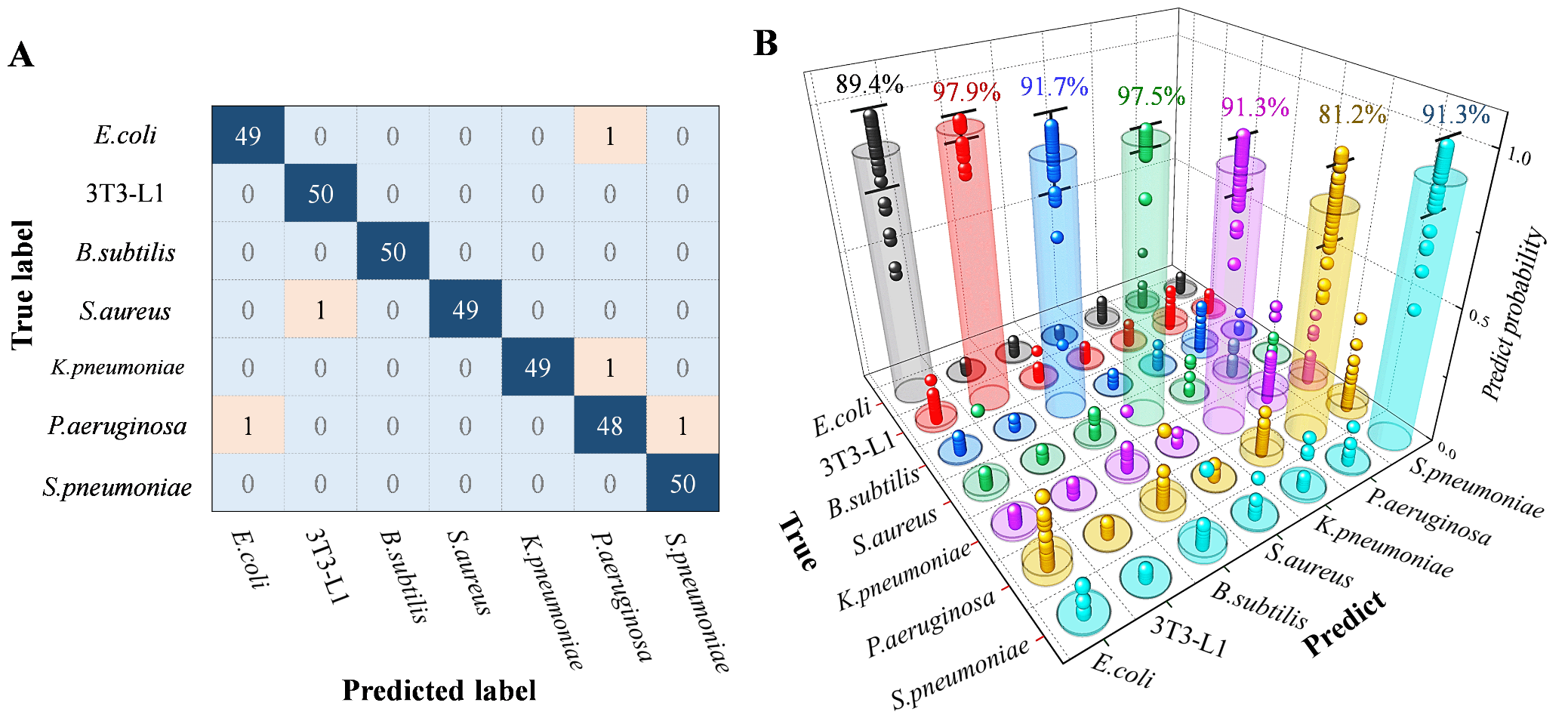


**Figure S11.** A Single Prediction on the Bacterial Spectra without Lysis Treatment. A) Confusion matrix showing the classification results, with 5 misclassified samples. B) Prediction probability matrix visualized as a 3D scatter plot with bars, illustrating the average probability distribution of the predictions. As observed, the recognition accuracy and robustness of the pre-lysis bacteria were significantly reduced, except for the 3T3-L1 cells.

**
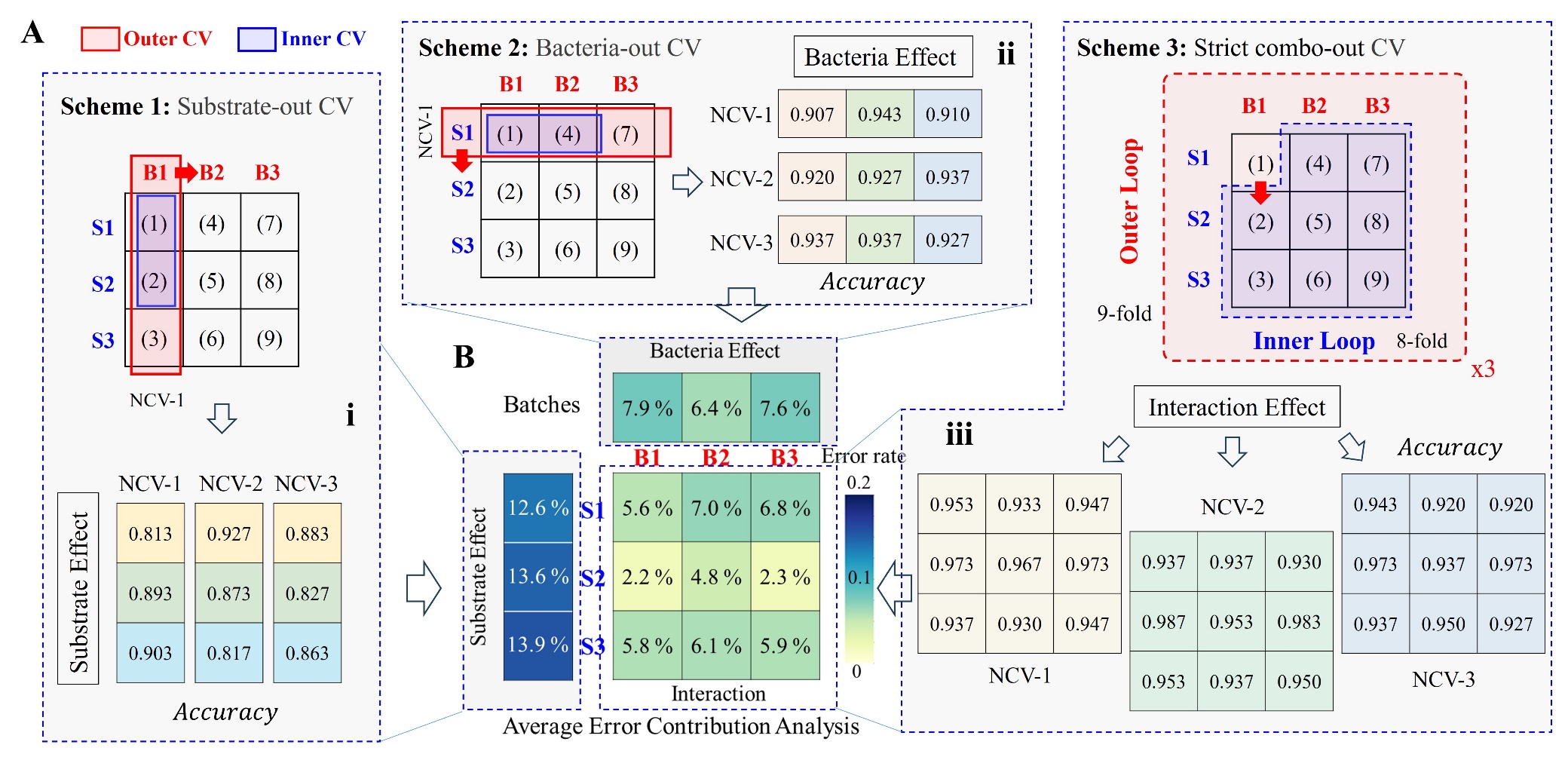
**

**Figure S12.** Outer‐Loop Test Results and Mean Error Across Three Nested CV Schemes. A) Test accuracies (%) for each held-out fold in the three nested cross-validation schemes. B) Mean test error heatmap for each substrate batch (S1–S3, Left bar heatmap), bacterial batch (B1–B3, Top bar heatmap) and substrate–bacteria combination (Central 3×3 heatmap). Color scales indicate error rate. All outer-loop test sets were strictly held out from any hyperparameter tuning.


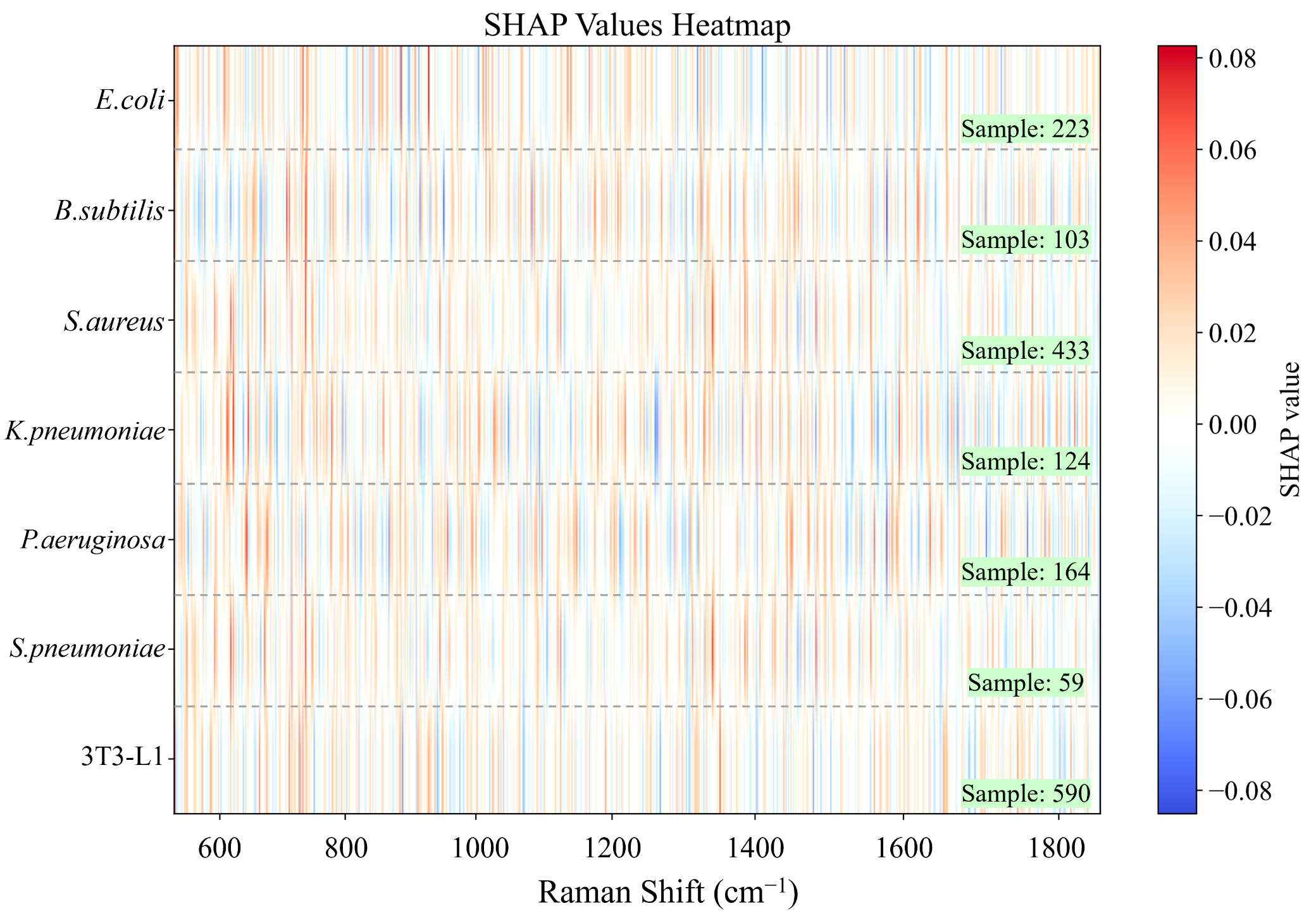


**Figure S13.** SHAP Values Heatmap for Raman Shift Contributions Across Seven Sample Types. This heatmap presents the SHAP values for Raman shift regions (600–1800 cm⁻¹) across seven different classes: *E. coli*, *B. subtilis*, *S. aureus*, *K. pneumoniae*, *P. aeruginosa*, *S. pneumoniae*, and 3T3-L1. Each row corresponds to a sample type, with the right-hand labels denoting individual spectra (e.g., Sample: 223). Positive SHAP values (red) represent Raman shifts that contribute positively to the model's prediction, while negative values (blue) indicate shifts with negative contributions. The map emphasizes key spectral features shared across multiple classes, as well as unique signatures that distinguish bacterial species from the 3T3-L1 cell line. This visualization provides critical insights into the spectral features driving bacterial classification.

***Enhancing Clinical Classification Accuracy via a Weighted Probability Algorithm***

To further improve the classification reliability for clinical samples, a weighted probability algorithm based on prediction confidence was applied. For each spectrum, the weight $W_{i}$​ was determined by the prediction probability $P_{i}$​ ​, with higher prediction probabilities corresponding to higher weights. The weighted probability $P_{weighted}$​ was then calculated as follows:

$P_{weighted}=\frac{\sum_{i=1}^{n} P_{i}\cdot W_{i}}{\sum_{i=1}^{n} W_{i}}$​

where $P_{i}$ represents the predicted probability of each individual Raman spectrum, and $W_{i}$​ is the weight assigned based on the confidence of the prediction. The clinical decision-making is based on the bacterial species with the highest weighted probability, ensuring a more reliable and accurate classification by emphasizing the measurements with greater confidence.


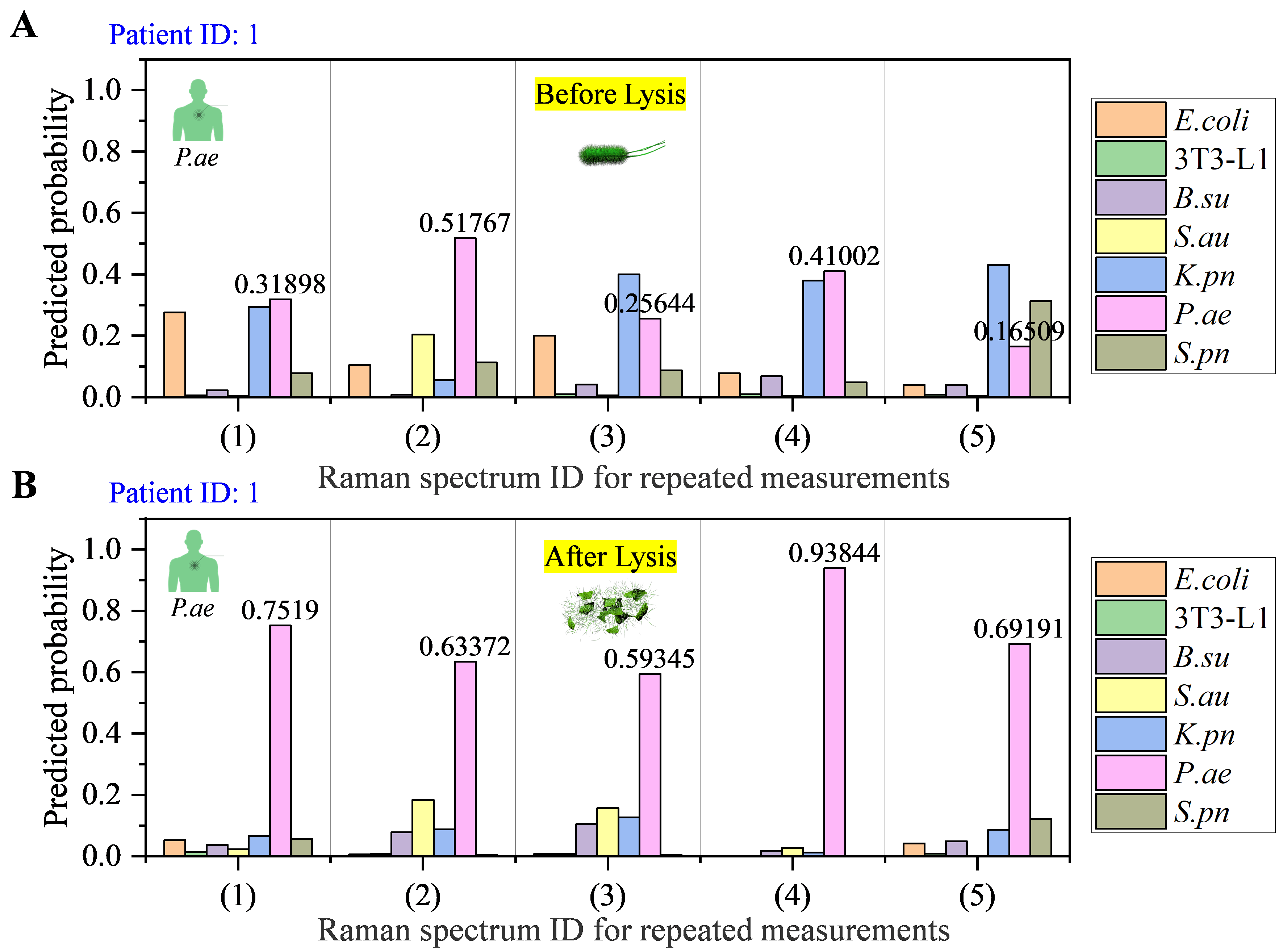


**Figure S14.** Detailed Prediction Analysis for Clinical Sample No.1 Before and After Lysis. A) Prediction probability distribution for five repeated measurements before lysis, showing high variability and reduced classification accuracy. B) Prediction probability distribution for five repeated measurements after lysis, highlighting improved accuracy and robustness.

For Patient No.1, the weighted probability algorithm was applied to the five individual Raman spectra obtained before lysis (Figure S14A) and after lysis (Figure S14B) for *P. aeruginosa* (*P.ae*). The weighted probability for *P.ae* before lysis was 0.3782, indicating low confidence and variability across the repeated measurements. In contrast, after lysis, the weighted probability for *P.ae* increased to 0.7449, demonstrating significantly improved consistency and reliability.

These results highlight the importance of the lysis process in enhancing prediction accuracy and robustness. The clinical decision-making process should be based on the bacterial species with the highest weighted probability. In this case, after lysis, the higher weighted probability for *P.ae* suggests a more confident identification of *P. aeruginosa*, providing a more reliable and accurate classification. This further emphasizes the potential of the lysis step in improving diagnostic outcomes, particularly in clinical settings where rapid and accurate pathogen identification is critical.


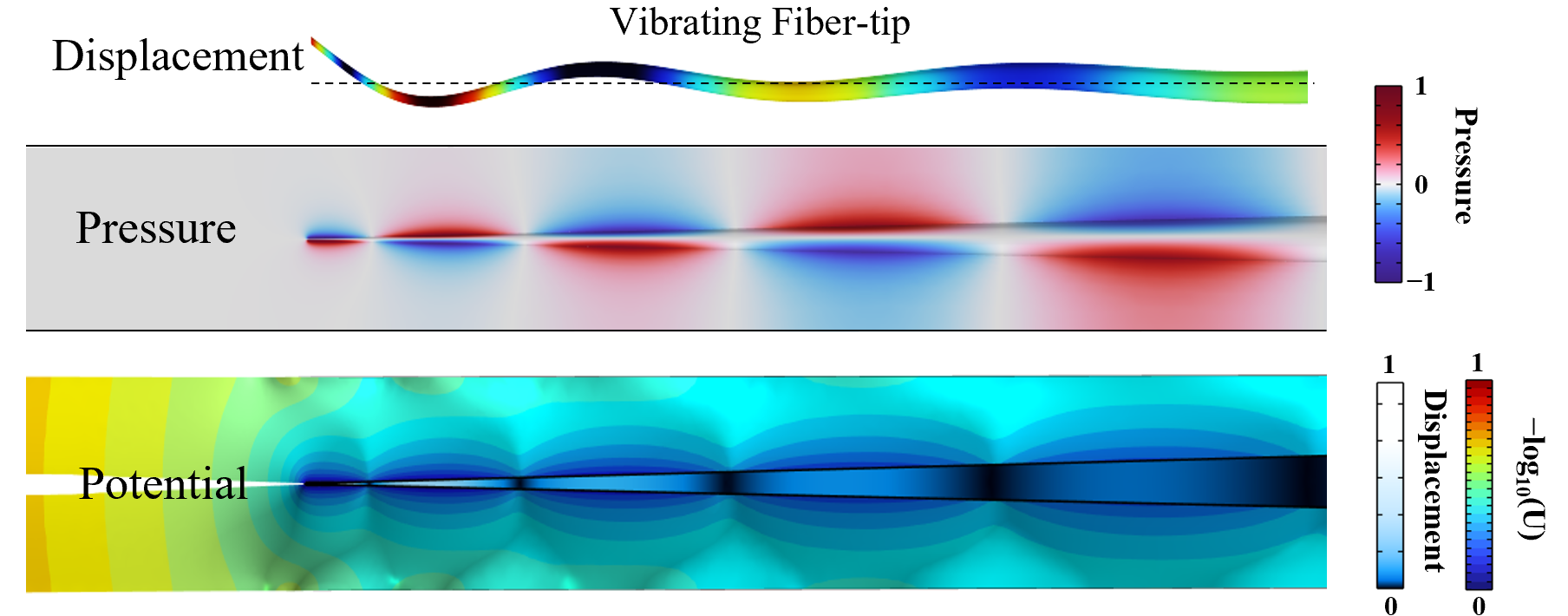


**Figure S15.** Simulation of the two-dimensional cross-sectional distribution of acoustic pressure and acoustic potential induced by the vibrating fiber tip. The maximum vibration amplitude corresponds to regions of high acoustic pressure, which are also the locations of the acoustic potential minima. This indicates that the strongest vibration areas coincide with the regions of highest acoustic radiation force.


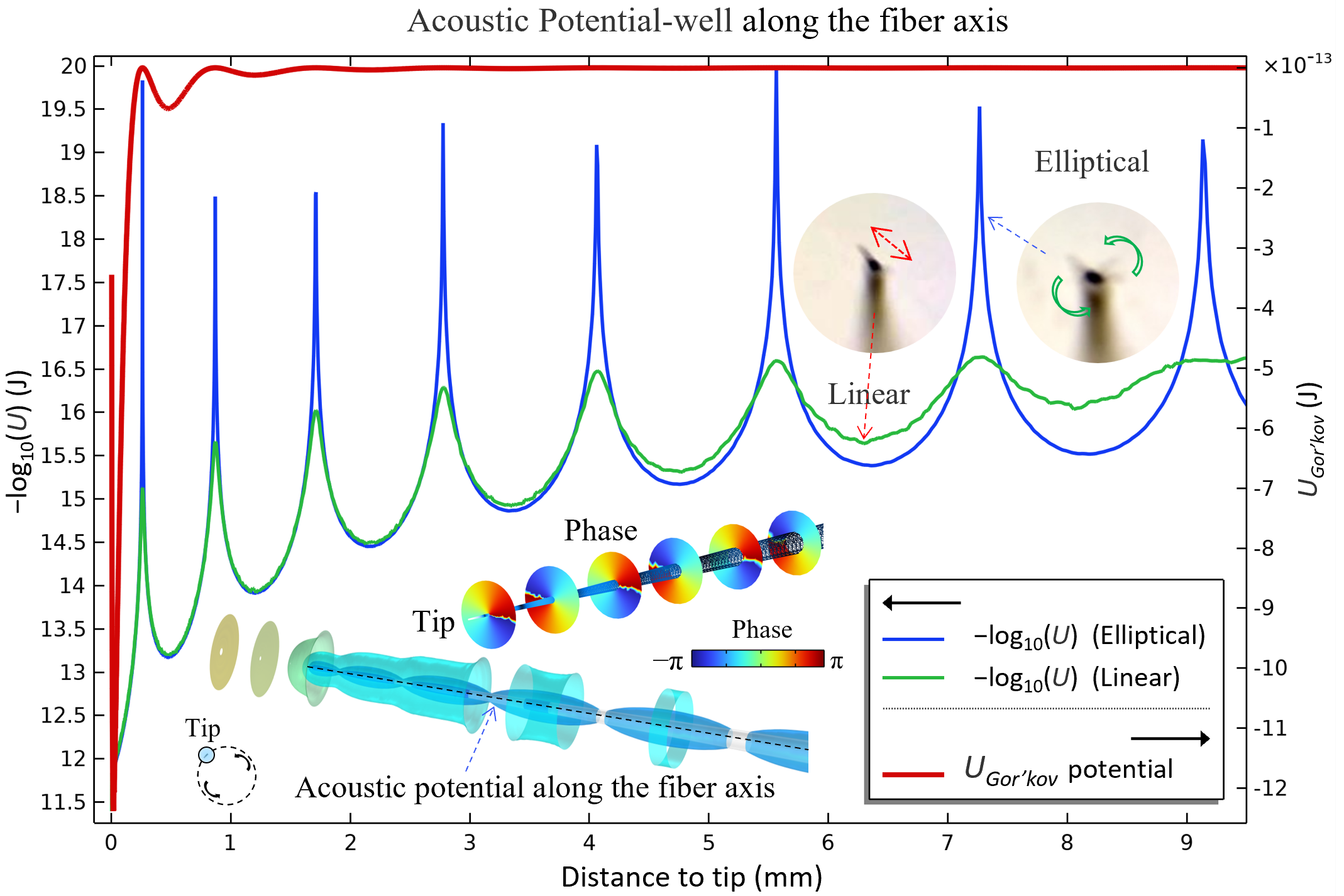


**Figure S16.** Distribution curves of the acoustic potential wells along the fiber axis (linear and circular-polarized vibration modes). A significant gradient of acoustic potential is observed near the fiber tip. After logarithmic scaling for visualization, the regions of acoustic potential minima are clearly revealed, demonstrating the particle trapping capability at these positions.

**
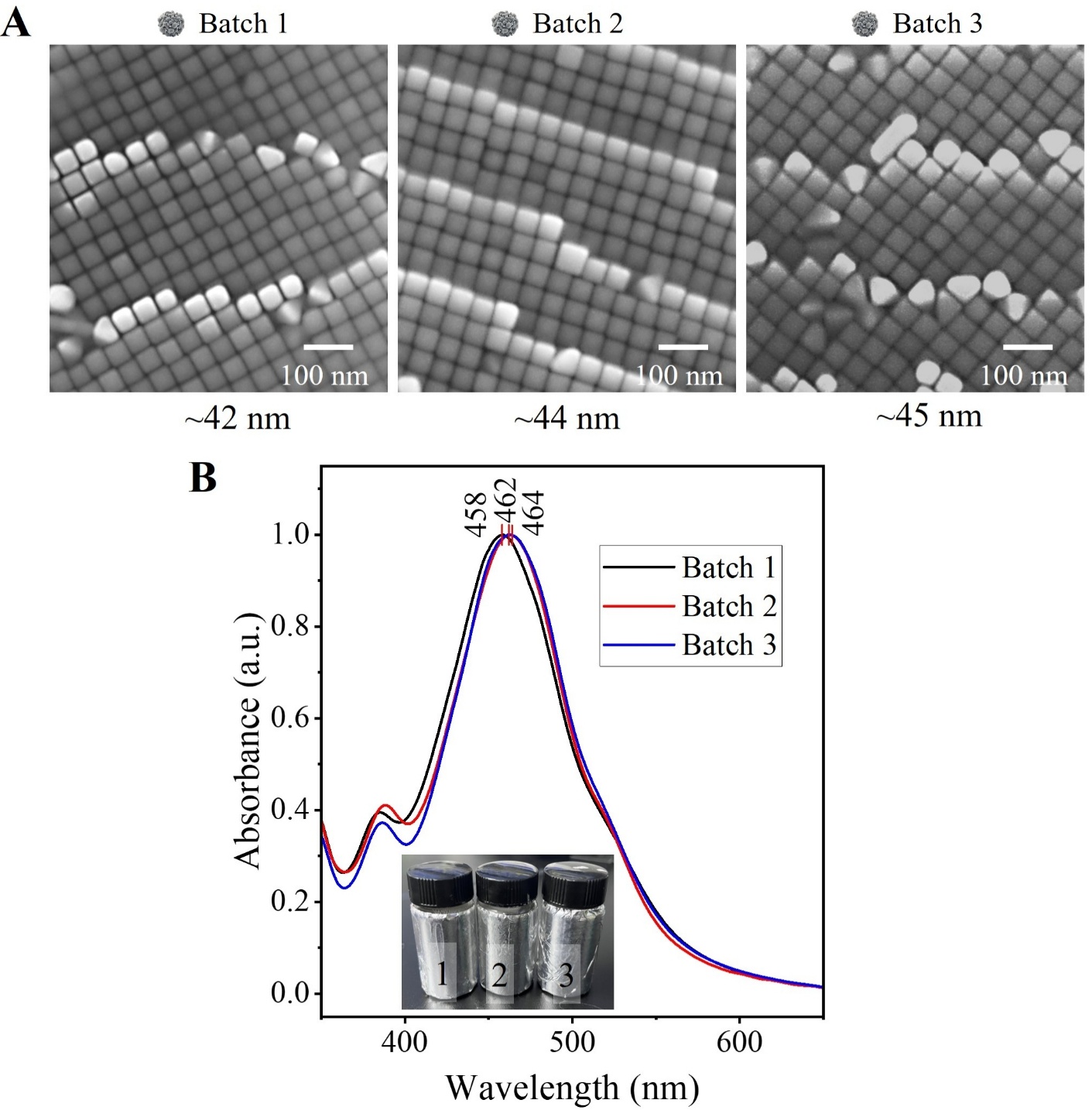
**

**Figure S17.** Characterization of three independently prepared batches of silver nanoparticles. A) Representative SEM micrographs of Batches 1–3 (left to right), showing well‐defined, uniformly spaced cubic nanoparticles with mean edge lengths of approximately 42 nm (Batch 1), 44 nm (Batch 2), and 45 nm (Batch 3). Scale bars: 100 nm. B) Normalized UV–Vis extinction spectra (n = 5 independent measurements per batch) illustrating the main localized surface plasmon resonance (SPR) peaks centered at 458 nm (Batch 1, black), 462 nm (Batch 2, red), and 464 nm (Batch 3, blue).


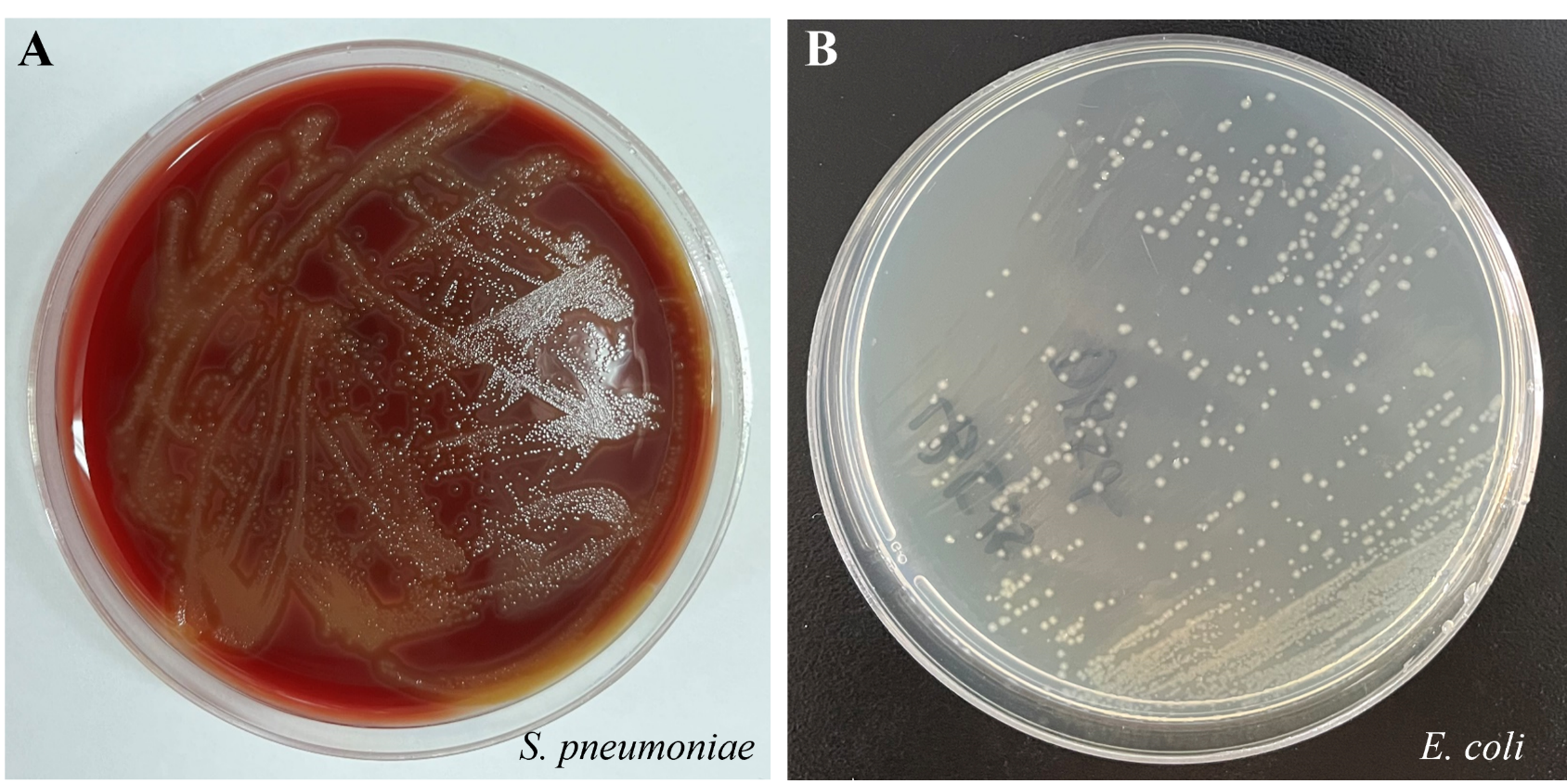


**Figure S18.** Images of partial bacterial culture on media. A) *Streptococcus pneumoniae* cultured on a blood agar plate. B) *Escherichia coli* cultured on an LB solid medium.


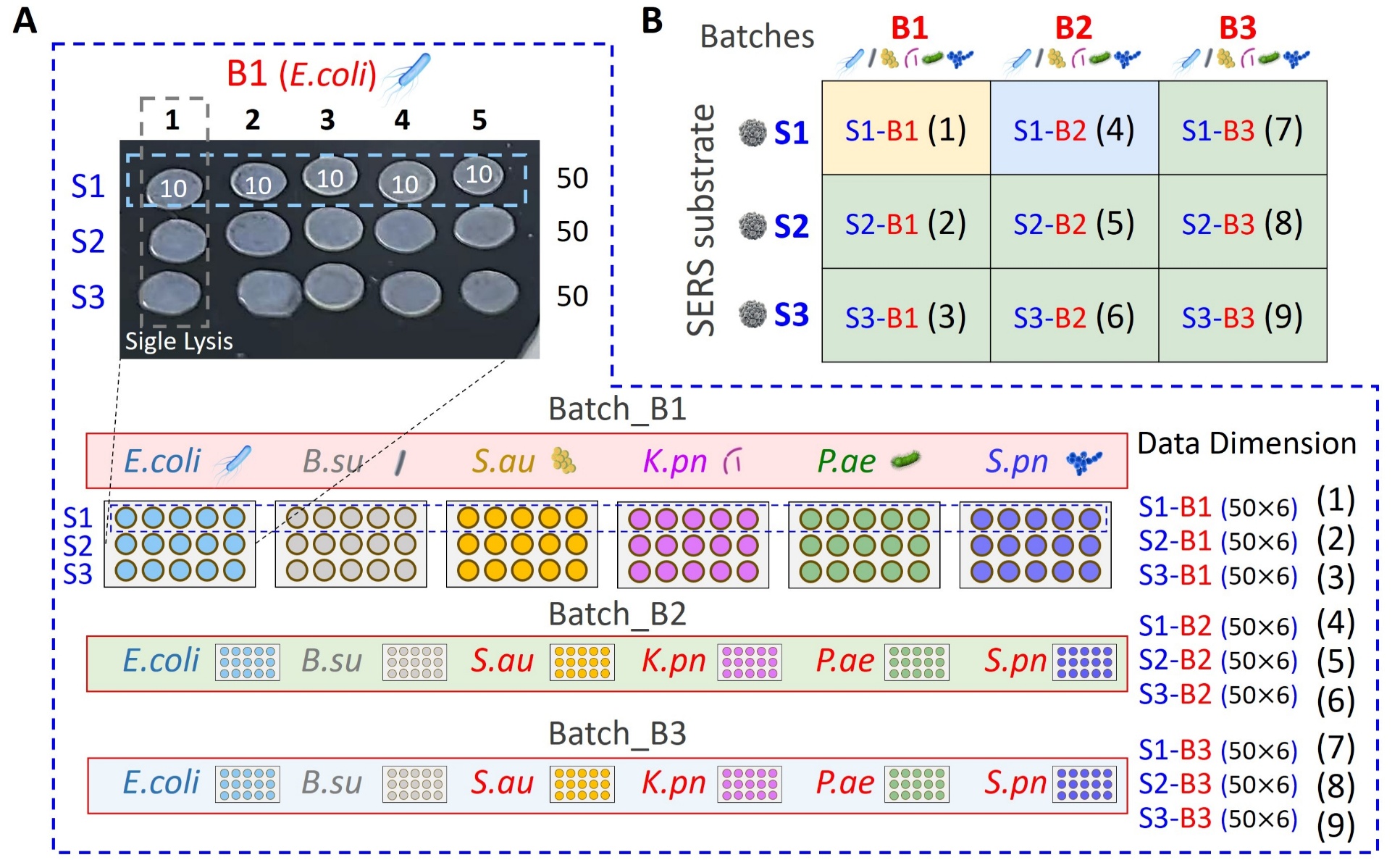


**Figure S19.** Experimental design for independent substrate–bacteria data collection. A) Workflow for a single bacterial batch (e.g., B1 = *E. coli*). Five technical replicates of lysate (15 Vpp, 60 s acoustofluidic lysis) were prepared, then each lysate (15 μL) was split into three ~5 μL aliquots and deposited onto three independently fabricated silver‑nanoparticle SERS substrates (S1, S2, S3). After air‑drying, ten SERS spectra were acquired per substrate replicate, yielding 50 spectra per species per substrate batch. B) Schematic of the 3×3 substrate–bacteria combination matrix (S1-B1 to S3-B3). Each combination (e.g., S1-B1) includes six bacterial species, with 50 spectra per species, resulting in a cumulative dataset of 2,700 spectra (50 spectra × 6 species × 9 combinations).


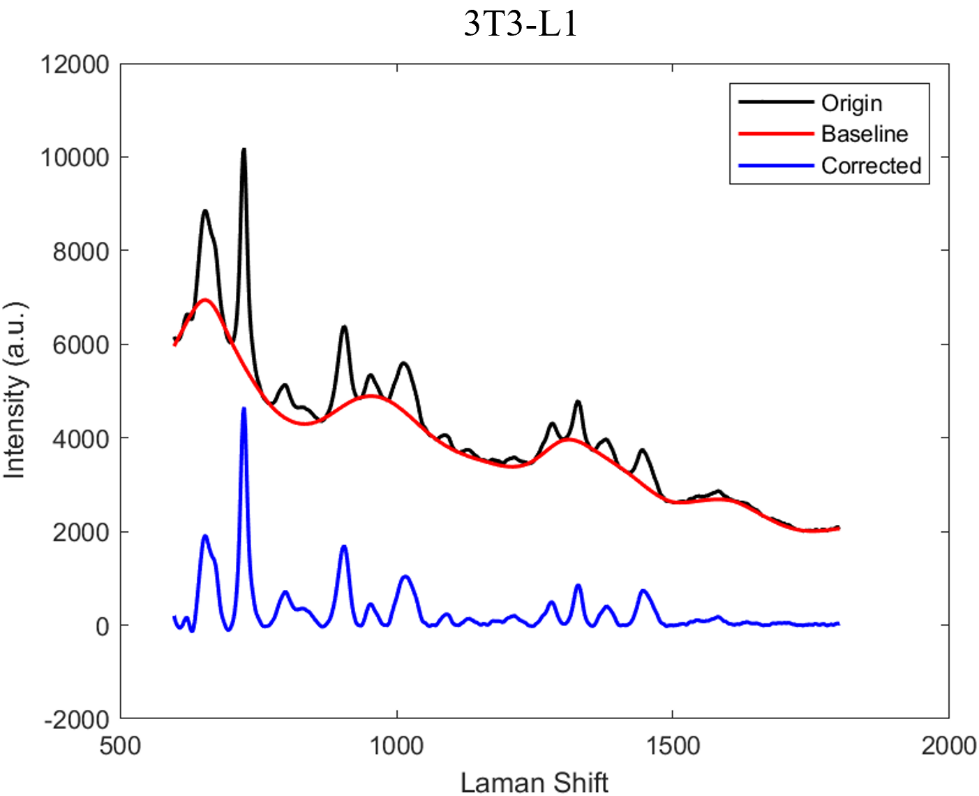


**Figure S20.** Raman spectra showing the original signal (black), baseline estimated by adaptive iterative reweighting penalized least squares (airPLS) (red), and the baseline-corrected signal (blue). Baseline correction was applied to remove fluorescence and other background signals, enhancing the visibility of characteristic Raman peaks for improved analysis.

**
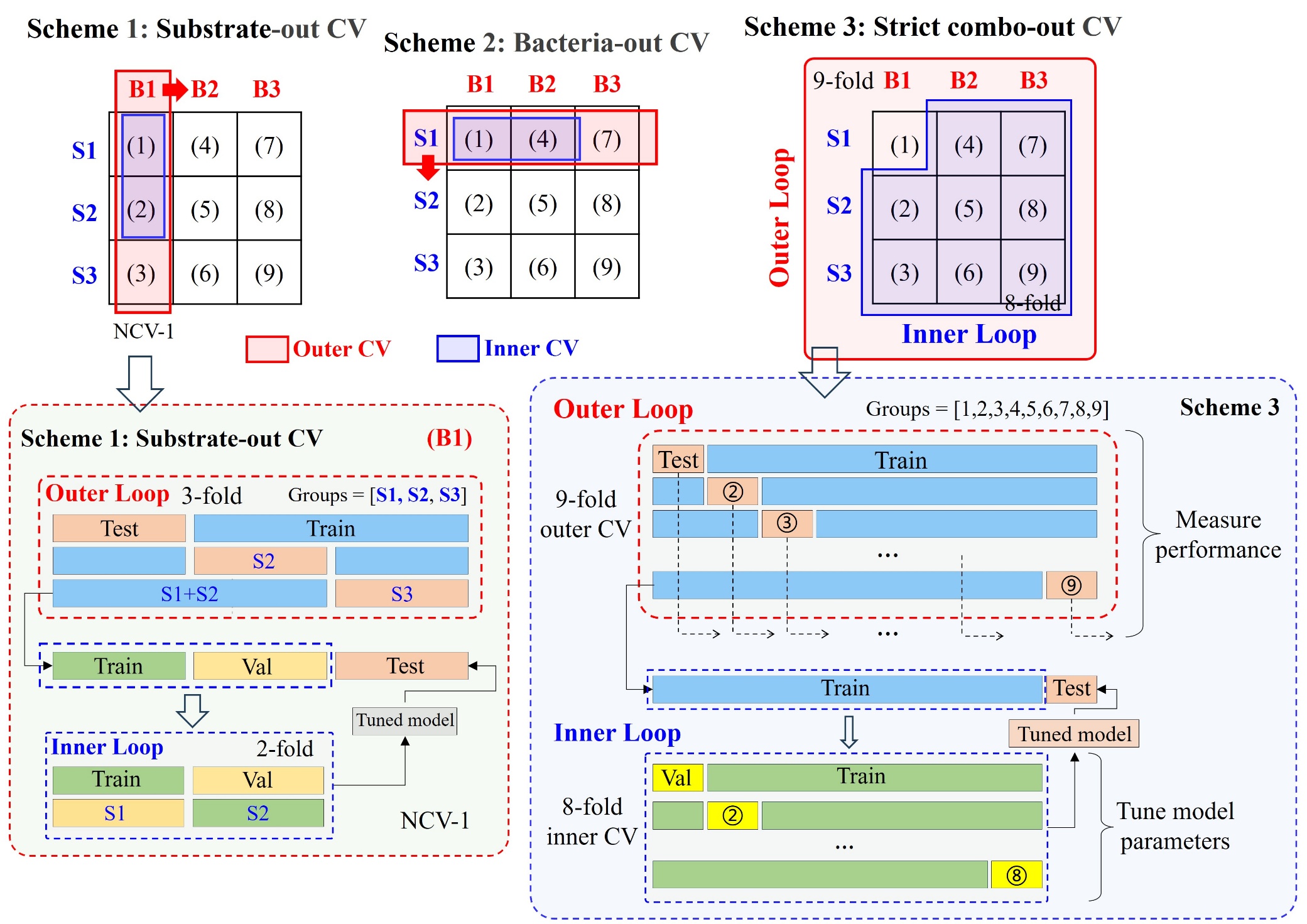
**

**Figure S21.** Strict Nested Cross-Validation (NCV) with Three Independent Validation Schemes. The schematic illustrates a rigorous NCV framework to mitigate data leakage and overfitting. *Top panel*: Simplified overview of three NCV schemes, featuring an outer CV loop for performance evaluation and an inner CV loop for hyperparameter optimization. *Lower panels*: Detailed workflows for Substrate-out CV (Scheme 1) and Strict Combo-out CV (Scheme 3).


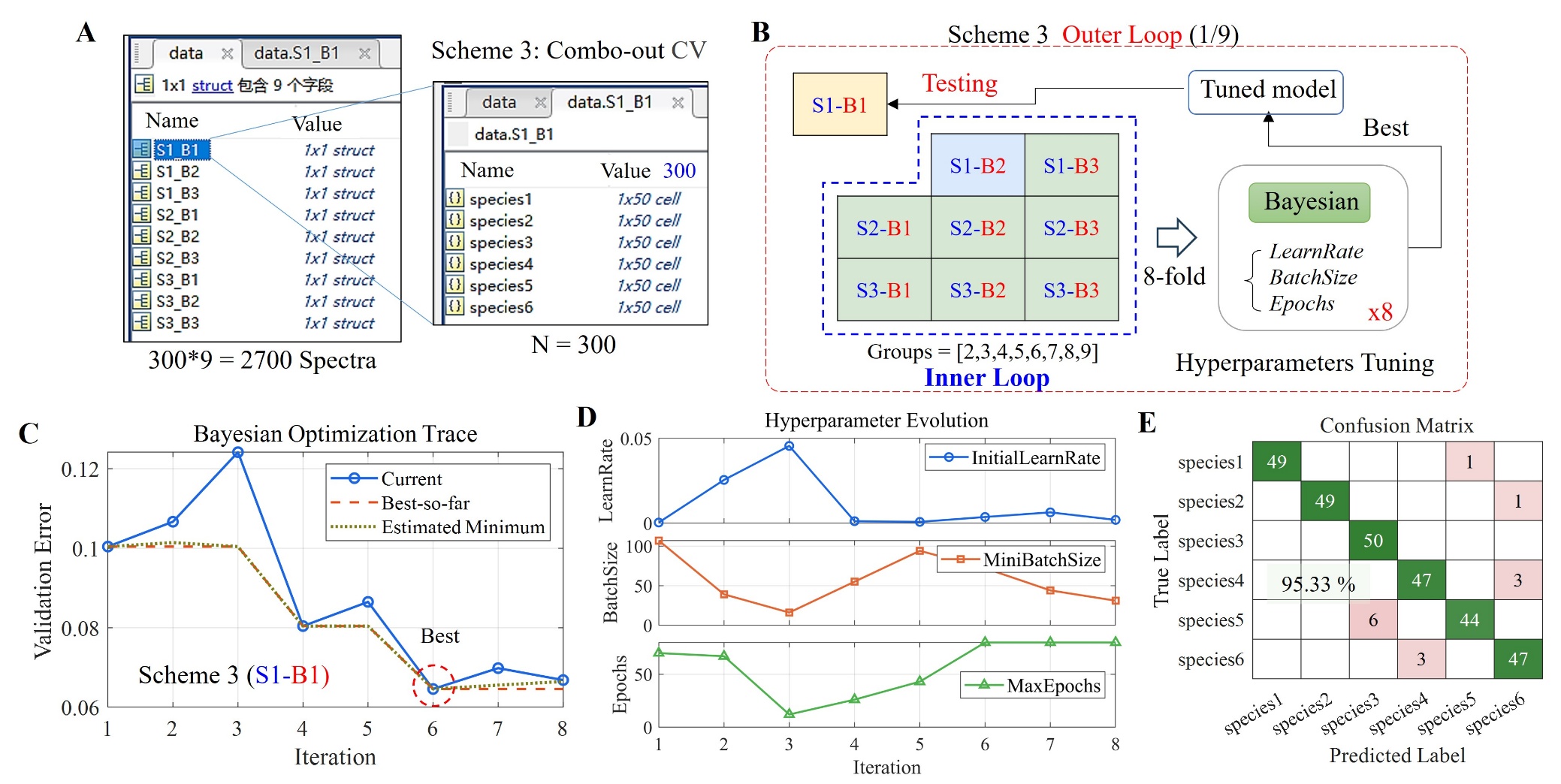


**Figure S22.** Inner-Loop Hyperparameter Optimization Process for the S1–B1 Outer Test Fold. A) Data structure in MATLAB: Left panel shows 9 substrate-bacteria combinations (300 spectra per combination, totaling 2,700 spectra). The zoomed-in panel (right) highlights the S1-B1 combination, comprising 6 species with 50 spectra each. B) Schematic of the S1–B1 outer‑loop iteration under Scheme 3 (combo‑out CV), the remaining 8 combinations serve as the inner-loop training/validation sets. Bayesian optimization was employed to tune three hyperparameters: initial learning rate, mini-batch size, and maximum epochs. C) Validation error traces over 8 iterations of Bayesian optimization. The blue circles, red dashed, and green dotted lines indicate the current validation error, best-so-far minimum, and estimated minimum error, respectively. D) Evolution of hyperparameter values during optimization: i) *InitialLearnRate* (blue), ii) *MiniBatchSize* (orange), and iii) *MaxEpochs* (green). The optimal parameters (6th iteration) selected are InitialLearnRate = 0.003687, MiniBatchSize = 72, and MaxEpochs = 80. E) Confusion matrix on the held‑out S1–B1 test set using the optimized model, yielding a classification accuracy of 95.33%. Species1 to Species6 represent *E. coli*, *B. subtilis*, *S. aureus*, *K. pneumoniae*, *P. aeruginosa*, and *S. pneumoniae*, respectively.


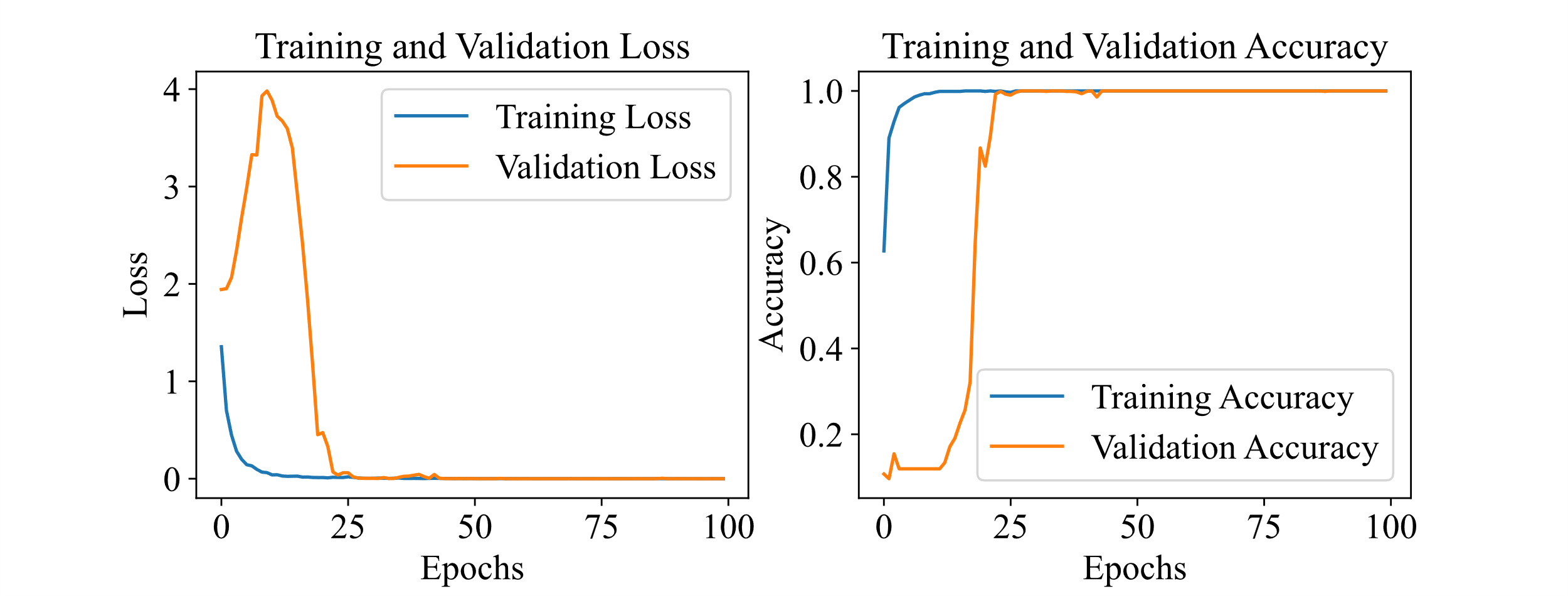


**Figure S23.** Training and validation loss and accuracy curves of the 1D-ResNet model over 100 epochs. The left plot shows the Training and Validation Loss: The training loss decreases steadily, approaching near-zero values, indicating effective learning. The validation loss, after some fluctuation in early epochs, also converges to a similarly low level, with no signs of overfitting. This is evident as both losses decrease concurrently, showcasing strong generalization. The right plot depicts Training and Validation Accuracy: Both training and validation accuracy improve rapidly, stabilizing at nearly 100% after approximately 25 epochs. This indicates the model's ability to accurately classify both training and unseen validation data, achieving strong performance without overfitting.


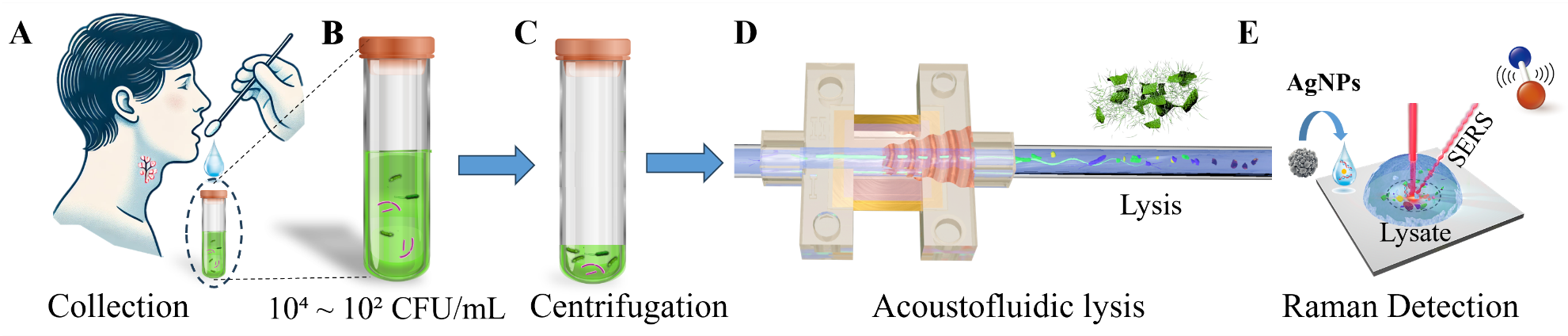


**Figure S24.** Workflow of Clinical Sample Processing and Raman Detection. A) Collection of clinical throat swab samples from patients. B) Sample suspension in a collection tube. C) Centrifugation to concentrate bacteria at the bottom of the tube. Removal of the supernatant, leaving a concentrated bacterial pellet. D) Introduction of the sample into a fiber-tip acoustic lysis device for bacterial disruption and Raman signal enhancement. E) Raman measurement.

**Table S1.** Tentative Band Assignments of the SERS Spectra.

| **Raman Shift (cm^−1^)** | | **Vibrational Assignment** | **Peak Assignment** |
| --- | --- | --- | --- |
| 620-650 | 624  643  650 | Aromatic ring skeleton, phenylalanine^[8]^  C-C twisting, tyrosine^[9]^  Ring breathing, guanine^[9]^ | Proteins  Proteins  Nucleic acids |
|  | 725  731 | Ring breathing, adenine^[9]^  Glycosidic ring mode^[10]^ | Nucleic acids  Polysaccharides |
| 780-800 | 780  787  800 | Ring breathing, thymine^[9]^  Ring breathing, cytosine^[11]^  Ring breathing, DNA/RNA^[12]^ | Nucleic acids |
|  | 841 | Cytosine, uracil, tyrosine^[11]^ | Nucleic acids |
| 840-860 | 840-860 | 1,4-glycosidic link stretching structure^[13]^  C-N stretching C-O-H bending^[14]^ | Polysaccharides  Peptidoglycan |
|  | 911-932 | C-C stretching^[15]^ | Proteins |
| 940-950 | 950 | CH_3_ symmetric stretching^[9]^ | Proteins (α-helix) |
|  | ~1000 | Ring breathing, phenylalanin^[16]^ | Proteins |
|  | 1024-1066 | C-C ring breathing, tyrosine/  phenylalanine^[15, 17]^ | Proteins |
|  | ~1100 | C-O stretching^[15]^ | Amides |
| 1220-1250 | 1228  1220-1235  1242 | PO_2−_ antisymmetric stretching  NH bending and CN stretching^[18]^  C-H in-plane/amide, thymine^[19]^ | DNA  Amide III, Proteins  Nucleic acids |
| 1300-1350 | 1300  1304  1321  1335 | In-plane twisting^[20]^  CH_2_ deformation^[20]^  CH_2_ deformation^[9, 20]^  CH₂ and CH₃ twisting^[18, 20]^ | Lipids  Lipids  Lipids  Proteins/Lipids |
|  | 1375~1380 | CH_2_ deformation  CH₃ symmetric deformation^[9]^ | Lipids |
|  | 1440~1450 | CH₂ and CH₃ deformation^[21]^ | Proteins/Lipids |
| 1460~1480 | 1472  1483 | CH₂ bending and CH₃ deformation^[18]^  Ring-breathing, guanine/adenine^[22]^ | Lipids  Nucleic acids |
| 1630~1650 | 1630-1650  1650 | Out-of-plane C–N stretching^[23]^  C=O stretching, amide I^[24]^ | Amino acid residues  Proteins (α-helix, β-fold) |
|  | 1660 | C=O stretching^[11]^ | Lipids |

Care should be taken when matching measured data with peaks in this table, as the peaks of the molecules may overlap with others.

**Table S2.** Comparative of Different Methods for Rapid Bacterial Identification.

| **Method** | **Detection Principle** | **Preprocessing Required** | | **Detection Time** | **Sensitivity** | **Advantages** | **Limitations** |
| --- | --- | --- | --- | --- | --- | --- | --- |
| **PCR/qPCR**  ^[25],[26],[27],[28]^ | Nucleic acid amplification | | Requires DNA extraction | 1-2 hours | Very High | Extremely sensitive | Time-consuming, specialized equipment |
| **MALDI-TOF**  ^[29],[30]^ | Mass spectrometry | | Requires bacterial colony isolation | ~15 min (after culturing) | High | Gold standard for clinical ID | Requires culture, expensive equipment |
| **Traditional SERS + ML**  ^[31],[32]^ | SERS + ML | | Requires bacterial culture | ~1-2 hours | Medium-High | Cost-effective, label-free | Requires bacterial preculture |
| **SERS Chip + ML**^[33]^ | SERS chip + ML | | Minimal | ~25 min | High | Label-free, flexible substrate | Requires optimized SERS chip fabrication |
| **Acoustic Bioprinting + SERS + ML**^[34]^ | Acoustic bioprinting + SERS + AI classification | | Requires sample digitization | ~1 hour | High | Suitable for liquid samples, high-throughput | Requires complex acoustic setup |
| **This Work (Acoustofluidic + SERS + ML)** | Acoustofluidic lysis + SERS + AI classification | | Minimal (Potential for direct clinical application) | <15 min | High | Rapid, label-free, high sensitivity | Requires specific substrates |

**Table S3:** The Key Material Parameters Used in Numerical Simulations.

| **Parameter** | **Quartz (Fiber)** | **Water (Fluid)** |
| --- | --- | --- |
| Density, ρ (kg/m³) | 2650 | 1000 |
| Sound Velocity, c (m/s) | 5700 | 1500 |
| Young's Modulus, E (GPa) | 73.1 | — |
| Poisson's Ratio, ν | 0.17 | — |
| Dynamic Viscosity, μ (Pa*s) | — | 1.0 × 10^-3^ |

**Supporting Videos**

**Video S1:** **Dynamic Evolution of Fiber-tip Vibration Modes.** The video consists of two segments. The first segment shows the vibration pattern as the driving frequency decreases from 9.80 kHz to 9.45 kHz. The second segment illustrates the changes in the vibration pattern with frequency adjustments in 10 Hz increments.

**Video S2:** **Simulations and Experiments: Vibrations and Acoustic Streaming Induced at the Fiber Tip.** The video consists of two segments. The first segment shows simulation results, illustrating the instantaneous velocity at the fiber tip under four vibration modes and the resulting wake effects. The second segment shows experimental results, displaying the acoustic streaming and the streaming patterns at the fiber tip under the same four vibration modes, which closely match the simulation outcomes.

**Video S3: Enrichment of Particles and Bacteria within a Capillary under Elliptical-Polarized (Torsional Vibration) Mode along the Fiber-tip.** The video consists of seven segments. The first segment shows the enrichment of 20 μm and 5 μm PS particles under bright-field illumination from two perspectives (top and side views). The second segment shows the enrichment of 2 μm fluorescent particles under dark-field illumination from two perspectives (front and side views). Segments three to seven respectively demonstrate the enrichment processes of 200 nm PS particles, 50 nm silver nanoparticles, *E. coli*, and *S. aureus*.

**Video S4:** Morphological changes of cells before and after acoustofluidic lysis.

**References**

[1] C. A. Lane, *J Acoust Soc Am* **1955**, *27* (6), 1082, <https://doi.org/10.1121/1.1908126>.

[2] C. P. Lee, T. G. Wang, *J Acoust Soc Am* **1989**, *85* (3), 1081, <https://doi.org/10.1121/1.397491>.

[3] D. E. Hughes, W. L. Nyborg, *Science* **1962**, *138* (3537), 108, <https://doi.org/10.1126/science.138.3537.108>.

[4] T. Tandiono, D. S. W. Ow, L. Driessen, C. S. H. Chin, E. Klaseboer, A. B. H. Choo, S. W. Ohl, C. D. Ohl, *Lab Chip* **2012**, *12* (4), 780, <https://doi.org/10.1039/c2lc20861j>.

[5] W. B. Wang, Y. S. Chen, U. Farooq, W. P. Xuan, H. Jin, S. R. Dong, J. K. Luo, *Appl Phys Lett* **2017**, *110* (14), <https://doi.org/10.1063/1.4979788>.

[6] S. C. Wang, X. Q. Lv, Y. Su, Z. Y. Fan, W. H. Fang, J. Z. Duan, S. Zhang, B. J. Ma, F. Liu, H. D. Chen, Z. X. Geng, H. Liu, *Small* **2019**, *15* (9), <https://doi.org/10.1002/smll.201804593>.

[7] U. Farooq, X. F. Liu, W. Zhou, M. Hassan, L. L. Niu, L. Meng, *Sensor Actuat B-Chem* **2021**, *345*, <https://doi.org/10.1016/j.snb.2021.130335>.

[8] H. B. Zhou, D. T. Yang, N. P. Ivleva, N. E. Mircescu, R. Niessner, C. Haisch, *Anal Chem* **2014**, *86* (3), 1525, <https://doi.org/10.1021/ac402935p>.

[9] N. Paccotti, F. Boschetto, S. Horiguchi, E. Marin, A. Chiadò, C. Novara, F. Geobaldo, F. Giorgis, G. Pezzotti, *Biosensors-Basel* **2018**, *8* (4), <https://doi.org/ARTN> 131

10.3390/bios8040131.

[10] A. Sivanesan, E. Witkowska, W. Adamkiewicz, L. Dziewit, A. Kaminska, J. Waluk, *Analyst* **2014**, *139* (5), 1037, <https://doi.org/10.1039/c3an01924a>.

[11] J. De Gelder, K. De Gussem, P. Vandenabeele, L. Moens, *Journal of Raman Spectroscopy* **2007**, *38* (9), 1133, <https://doi.org/10.1002/jrs.1734>.

[12] F. Safir, N. Vu, L. F. Tadesse, K. Firouzi, N. Banaei, S. S. Jeffrey, A. A. E. Saleh, B. T. Khuri-Yakub, J. A. Dionne, *Nano Lett* **2023**, *23* (6), 2065, <https://doi.org/10.1021/acs.nanolett.2c03015>.

[13] M. Tahir, M. I. Majeed, H. Nawaz, S. Ali, N. Rashid, M. Kashif, I. Ashfaq, W. Ahmad, K. Ghauri, F. Sattar, I. Jawad, M. A. Ghauri, M. A. Anwar, *Spectrochim Acta A* **2020**, *237*, <https://doi.org/10.1016/j.saa.2020.118408>.

[14] N. P. Ivleva, M. Wagner, H. Horn, R. Niessner, C. Haisch, *Analytical and Bioanalytical Chemistry* **2009**, *393* (1), 197, <https://doi.org/10.1007/s00216-008-2470-5>.

[15] S. Y. Liu, Q. S. Hu, C. Li, F. R. Zhang, H. J. Gu, X. R. Wang, S. Li, L. Xue, T. Madl, Y. Zhang, L. Zhou, *Acs Sensors* **2021**, *6* (8), 2911, <https://doi.org/10.1021/acssensors.1c00641>.

[16] M. T. Alula, S. Krishnan, N. R. Hendricks, L. Karamchand, J. M. Blackburn, *Microchim Acta* **2017**, *184* (1), 219, <https://doi.org/10.1007/s00604-016-2013-2>.

[17] M. Chisanga, H. Muhamadali, D. I. Ellis, R. Goodacre, *Appl Spectrosc* **2018**, *72* (7), 987, <https://doi.org/10.1177/0003702818764672>.

[18] F. Moghtader, A. Tomak, H. M. Zareie, E. Piskin, *Artif Cell Nanomed B* **2018**, *46*, 122, <https://doi.org/10.1080/21691401.2018.1452251>.

[19] A. Nakar, A. Pistiki, O. Ryabchykov, T. Bocklitz, P. Rösch, J. Popp, *Analytical and Bioanalytical Chemistry* **2022**, *414* (4), 1481, <https://doi.org/10.1007/s00216-021-03800-y>.

[20] K. Czamara, K. Majzner, M. Z. Pacia, K. Kochan, A. Kaczor, M. Baranska, *Journal of Raman Spectroscopy* **2015**, *46* (1), 4, <https://doi.org/10.1002/jrs.4607>.

[21] W. E. Huang, M. Q. Li, R. M. Jarvis, R. Goodacre, S. A. Banwart, *Adv Appl Microbiol* **2010**, *70*, 153, <https://doi.org/10.1016/S0065-2164(10)70005-8>.

[22] K. Maquelin, C. Kirschner, L. P. Choo-Smith, N. van den Braak, H. P. Endtz, D. Naumann, G. J. Puppels, *J Microbiol Meth* **2002**, *51* (3), 255, <https://doi.org/10.1016/S0167-7012(02)00127-6>.

[23] J. A. Huang, M. Z. Mousavi, G. Giovannini, Y. Q. Zhao, A. Hubarevich, M. A. Soler, W. Rocchia, D. Garoli, F. De Angelis, *Angew Chem Int Edit* **2020**, *59* (28), 11423, <https://doi.org/10.1002/anie.202000489>.

[24] R. M. Jarvis, R. Goodacre, *Fems Microbiol Lett* **2004**, *232* (2), 127, <https://doi.org/10.1016/S0378-1097(04)00040-0>.

[25] M. J. Espy, J. R. Uhl, L. M. Sloan, S. P. Buckwalter, M. F. Jones, E. A. Vetter, J. D. C. Yao, N. L. Wengenack, J. E. Rosenblatt, F. R. Cockerill, T. F. Smith, *Clin Microbiol Rev* **2006**, *19* (1), 165, <https://doi.org/10.1128/Cmr.19.1.165-256.2006>.

[26] P. Kralik, M. Ricchi, *Front Microbiol* **2017**, *8*, <https://doi.org/ARTN> 108

10.3389/fmicb.2017.00108.

[27] S. J. Salipante, K. R. Jerome, *Clin Chem* **2020**, *66* (1), 117, <https://doi.org/10.1373/clinchem.2019.304048>.

[28] S. W. Lei, S. Chen, Q. P. Zhong, *Int J Biol Macromol* **2021**, *184*, 750, <https://doi.org/10.1016/j.ijbiomac.2021.06.132>.

[29] A. Croxatto, G. Prod'hom, G. Greub, *Fems Microbiol Rev* **2012**, *36* (2), 380, <https://doi.org/10.1111/j.1574-6976.2011.00298.x>.

[30] C. V. Weis, C. R. Jutzeler, K. Borgwardt, *Clin Microbiol Infec* **2020**, *26* (10), 1310, <https://doi.org/10.1016/j.cmi.2020.03.014>.

[31] S. Das, K. Saxena, J. C. Tinguely, A. Pal, N. L. Wickramasinghe, A. Khezri, V. Dubey, A. Ahmad, V. Perumal, R. Ahmad, D. N. Wadduwage, B. S. Ahluwalia, D. S. Mehta, *Acs Appl Mater Inter* **2023**, *15* (20), 24047, <https://doi.org/10.1021/acsami.3c00612>.

[32] D. Paria, K. S. Kwok, P. Raj, P. Zheng, D. H. Gracias, I. Barman, *Nano Lett* **2022**, *22* (9), 3620, <https://doi.org/10.1021/acs.nanolett.1c04722>.

[33] C. S. Ho, N. Jean, C. A. Hogan, L. Blackmon, S. S. Jeffrey, M. Holodniy, N. Banaei, A. A. E. Saleh, S. Ermon, J. Dionne, *Nat Commun* **2019**, *10* (1), 4927, <https://doi.org/10.1038/s41467-019-12898-9>.

[34] F. Safir, N. Vu, L. F. Tadesse, K. Firouzi, N. Banaei, S. S. Jeffrey, A. A. E. Saleh, B. P. T. Khuri-Yakub, J. A. Dionne, *Nano Lett* **2023**, *23* (6), 2065, <https://doi.org/10.1021/acs.nanolett.2c03015>.
